# Perceptual reorganization from prior knowledge emerges late in childhood

**DOI:** 10.1016/j.isci.2024.108787

**Published:** 2024-01-04

**Authors:** Georgia A. Milne, Matteo Lisi, Aisha McLean, Rosie Zheng, Iris I.A. Groen, Tessa M. Dekker

**Affiliations:** 1 Institute of Ophthalmology, University College London, EC1V 9EL London, UK; 2 Division of Psychology and Language Sciences, University College London, WC1H 0AP London, UK; 3 Department of Psychology, Royal Holloway, University of London, TW20 0EX London, UK; 4 Informatics Institute, University of Amsterdam, 1098 XH Amsterdam, the Netherlands

**Keywords:** Neuroscience, Developmental neuroscience, Psychology

## Abstract

Human vision relies heavily on prior knowledge. Here, we show for the first time that prior-knowledge-induced reshaping of visual inputs emerges gradually in late childhood. To isolate the effects of prior knowledge on perception, we presented 4- to 12-year-olds and adults with two-tone images – hard-to-recognize degraded photos. In adults, seeing the original photo triggers perceptual reorganization, causing mandatory recognition of the two-tone version. This involves top-down signaling from higher-order brain areas to early visual cortex. We show that children younger than 7–9 years do not experience this knowledge-guided shift, despite viewing the original photo immediately before each two-tone. To assess computations underlying this development, we compared human performance to three neural networks with varying architectures. The best-performing model behaved much like 4- to 5-year-olds, displaying feature-based rather than holistic processing strategies. The reconciliation of prior knowledge with sensory input undergoes a striking age-related shift, which may underpin the development of many perceptual abilities.

## Highlights

• Photo cues reliably cause perceptual reorganization of two-tone images in adults
• Perceptual reorganization does not emerge until at least 7–9 years of age
• CNNs’ two-tone recognition is low, and correlates with that of young children

## Graphical abstract

**Figure:**
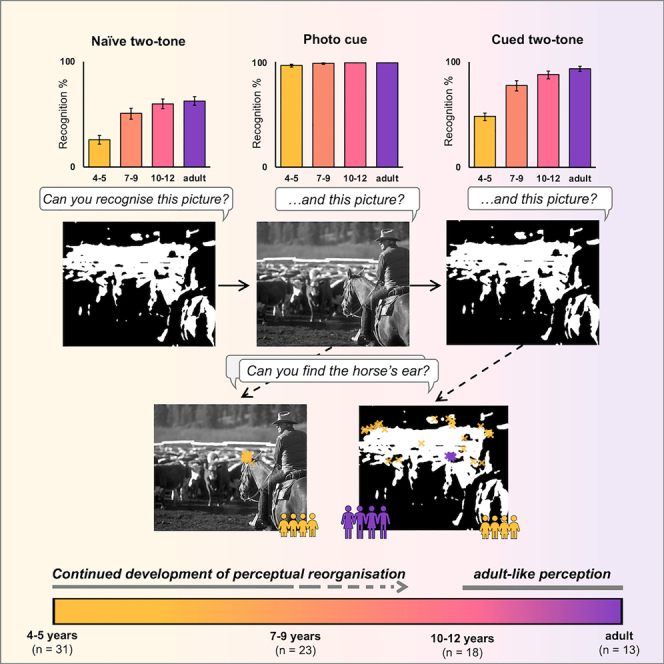

## Introduction

The mature human visual system is unique in its ability to accurately recognize objects across a wide range of viewing circumstances. Reaching this level of robustness in object recognition poses a major challenge to computer vision algorithms^1^^,^^2^^,^^3^^,^^4^ and to developing humans. Though infants’ perception of simple shapes is invariant to orientation soon after birth,^5^ the ability to recognize and classify more complex objects develops gradually after the first few months of life.^6^^,^^7^ This ability likely requires extended visual exploration of objects,^8^ and incorporates semantic labeling of visual categories as infants begin to learn language.^9^^,^^10^ By 1–2 years old, infants demonstrate an understanding of the relationship between the visual and lexical categories of familiar objects^11^; however, recognition of these objects under challenging viewing conditions, such as clutter, noise, abstraction, and unusual lighting or orientations, does not become adult-like until at least 10 years old.^12^^,^^13^^,^^14^ Current predictive coding models of human vision assign a crucial role to prior knowledge, delivered via top-down pathways in the brain, for parsing ambiguous or cluttered images.^15^^,^^16^^,^^17^^,^^18^ Meanwhile, structural and functional MRI measures of long-range neural connectivity that may mediate these feedback signals have been shown to increase continuously over the first decade of life.^19^^,^^20^ We therefore predict that effective perception of hard-to-recognize objects may develop gradually as long-range neural connections are established across childhood, improving knowledge-based parsing. To track this perceptual development, we asked children aged 4 to 12 years old to identify 'two-tone' image stimuli, which allow us to disentangle the presence of object knowledge from the ability to use this knowledge to inform perceptual inference.

Following their conception by artist Giorgio Kienerk (1869–1948) and introduction to psychology by Craig Mooney,^21^ two-tone images have offered a famous example of the importance of prior knowledge for recognition. By subjecting grayscale images to Gaussian smoothing and binarization, visual information is reduced and object boundaries are ambiguated in the resulting black-and-white two-tone.^22^ These images are not easily recognizable when viewed naively, but have the intriguing property that once additional cues are made available, recognition becomes near-mandatory in subsequent viewings. This process, referred to as perceptual reorganization, is thought to involve feedback to low-level visual cortex from higher-order brain areas, including the lateral occipital and prefrontal cortex.^23^^,^^24^^,^^25^^,^^26^^,^^27^^,^^28^ Pharmacological interference of top-down pathways reduces the similarity of responses in primary visual cortex (V1) to two-tone and grayscale images, suggesting a causal role of these feedback connections in perceptual reorganization.^29^ This top-down information processing may operate by enhancing visual sensitivity to low-level image features of the two-tones computed in V1, such as orientation and edge information.^30^ This is consistent with Bayesian models of perceptual inference, where priors are propagated to lower areas via top-down processes and combined with bottom-up information to shape perception.^17^^,^^31^

Two-tone image recognition thus provides a well-controlled paradigm for studying how the use of prior knowledge to parse ambiguous images develops. Though Mooney himself already showed that the ability to naively recognize two-tones of faces is impaired during childhood and improves with age,^21^ less is known about children’s ability to recognize non-facial two-tone stimuli, and even less so about the development of perceptual reorganization. In their book chapter on binocular perception, Kovács and Eisenberg^32^ hypothesized this ability may also be impaired in young children. In a 2007 conference paper,^33^ Yoon and colleagues then reported low accuracy locating object features on two-tones in a small sample of 3- to 4-year-olds, despite them having seen the original photo, in line with reduced perceptual reorganization. Thus, across childhood there may be drastic changes in how prior knowledge guides perceptual inference about ambiguous images, which may have broad-reaching implications for the development of object recognition. However, this is currently underappreciated in the literature, and a characterization of two-tone image perception across childhood that systematically tests this is currently lacking.

Here, we address this gap by testing perceptual reorganization and two-tone image processing throughout childhood in 72 four- to twelve-year-olds. Crucially, we use methodological and analytical approaches that systematically account for potential confounding factors besides the ability to use prior knowledge to parse two-tones that also change with age. These include object knowledge, task comprehension, and response execution. We explore which computational mechanisms may underlie shifts from child-like toward adult-like two-tone recognition by investigating image parsing and feature extraction strategies used at different ages. We compare human performance to that of convolutional neural networks (CNNs) to investigate the extent to which the architectural properties of these models, i.e., incorporation of feedback processing or computational depth, can emulate early stages of human visual development. We find striking differences in the way that young children, and CNNs, parse ambiguous two-tone images, whereby adult levels of prior-knowledge-driven perception do not develop until late childhood. As in computational object classifiers, we link children’s reduced recognition of two-tone images to a more featural-based processing strategy.

## Results

**Figure 1:**
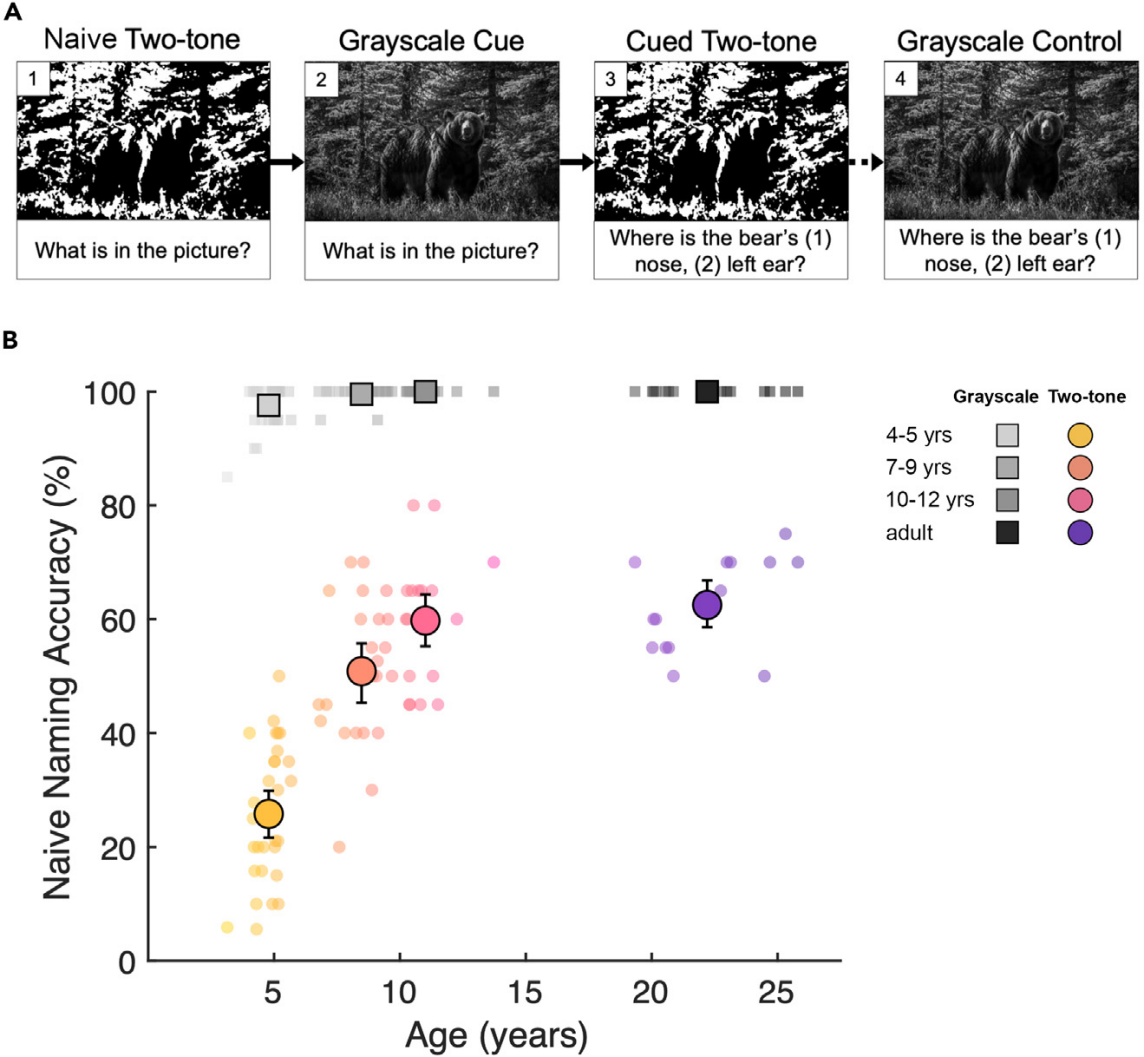
Experimental trial schema and naive two-tone recognition accuracy (A) An example trial for one image, stages 1 (naive two-tone naming), 2 (grayscale cueing and naming), and 3 (cued two-tone pointing) are sequential, and stage 4 (grayscale control pointing) occurs separately after the main task. (B) Naming accuracy of naive two-tones (colored circles; see stage 1 in A), and grayscale images (gray squares; see stage 2 in A). Small markers show participant means, large markers show age group means, and error bars show bootstrapped 95% confidence intervals.

4- to 5-year-olds (n = 31), 7- to 9-year-olds (n = 23), 10- to 12-year-olds (n = 18), and adults (n = 13) sequentially viewed two-tone images on a touchscreen before and after cueing with the corresponding grayscale image. In each trial, participants were asked to name the content of the naively viewed two-tone (Figure 1A, stage 1), after which the image transitioned into the corresponding grayscale to cue object knowledge, and participants were again asked to name the content to confirm they could recognize the image (Figure 1A, stage 2). To measure perceptual reorganization, this image transitioned back to the same two-tone and participants were asked to touch the locations of two characteristic object features (Figure 1A, stage 3). After completing trials for twenty two-tones and four Catch images (unsmoothed two-tones), participants were shown each grayscale image again and asked to touch the same target features that were prompted for the corresponding two-tone images (Figure 1A, stage 4).

### Accounting for age differences in grayscale recognition

To account for potential age differences in object knowledge, we first established that grayscale naming accuracy was high and matched at all ages (Figure 1B, gray squares, Χ^2^(3) = 3.58, p = 0.31). Nevertheless, trials in which grayscales were incorrectly named were excluded from all further analyses (total trials excluded for 4- to 5-year-olds = 15, 7- to 9-year-olds = 2, 10- to 12-year-olds and adults = 0).

### Age differences in naive two-tone recognition

In contrast to the accurate naming of the grayscale images at all ages, a logistic mixed-effects model (see STAR Methods for details) revealed that naive naming accuracy for the two-tone versions of these grayscales was lower, and improved substantially with age (Χ^2^(3) = 89.88, p = 2.2e-16; Figure 1B, colored circles). Wald tests on model coefficients revealed that 4- to 5-year-olds (z = −9.6; p < 2.0e-16) and 7- to 9-year-olds (z = −3.2; p < 1.38e-3) were less accurate than adults, while 10- to 12-year-olds performed similarly to adults (z = −0.70, p = 0.48). This age difference is unlikely due to failure to comprehend or comply with the task since performance for easily recognizable Catch images was high for all ages (see Figure S2A). Mean naming accuracy per image for naively viewed two-tones was correlated between all age groups (r_pearson’s_ > 0.7, p < 5.71e-4, see Figure S4 for all correlations). Together, this suggests that up to at least 7 to 9 years of age, recognition of familiar objects is more impaired by two-tone transformation than in adulthood, but that the features that make a two-tone hard to recognize at first sight remain qualitatively consistent across age.

### Age differences in perceptual reorganization

**Figure 2:**
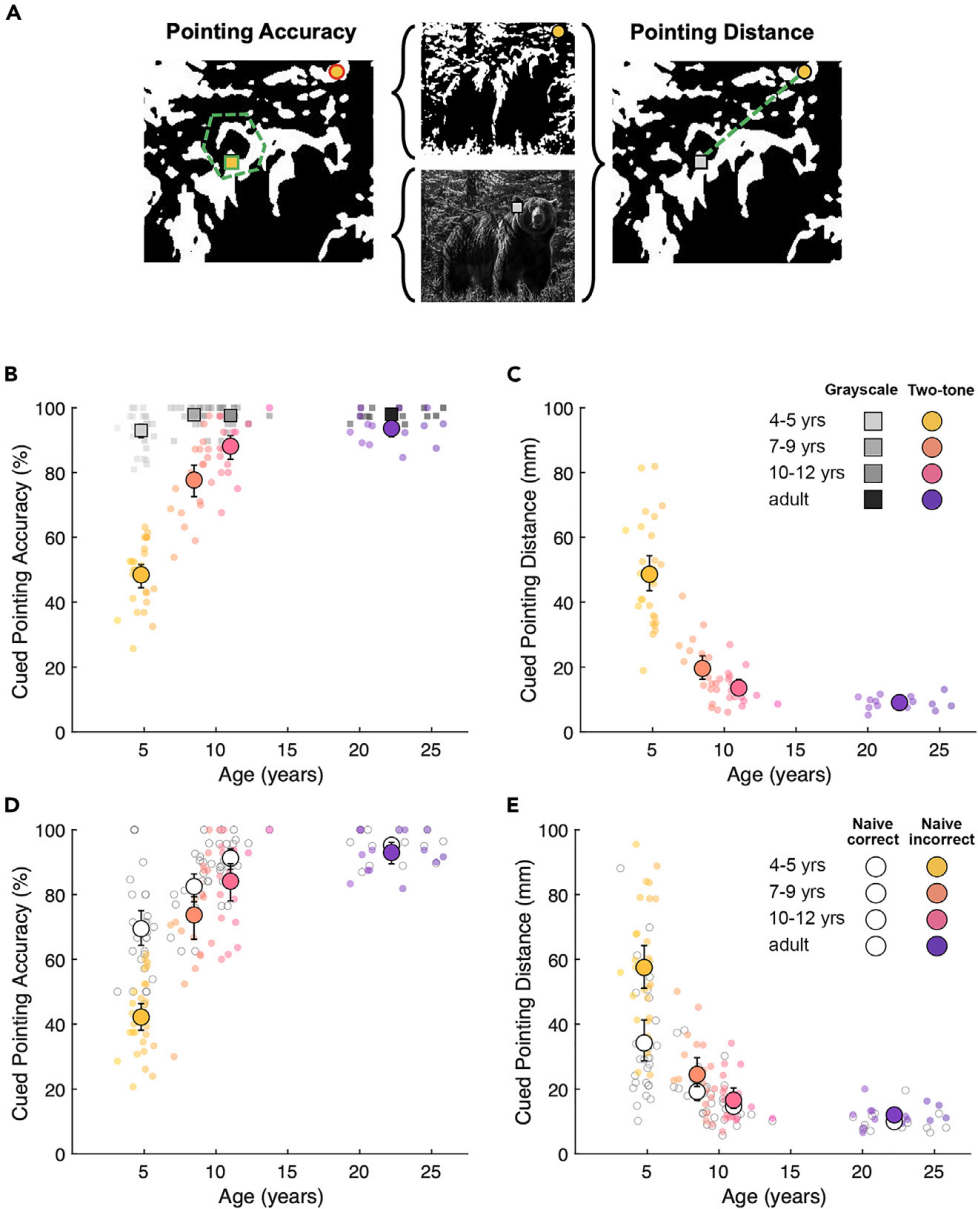
Cued two-tone recognition measured by pointing accuracy and distance (A) Circular and square markers show example two-tone and grayscale cued pointing responses for one of two targets, respectively. Touched points were scored via two methods; the dashed polygon shows the example 'correct' feature location used to determine 'pointing accuracy' (green outlined marker scored as correct and red outlined marker scored as incorrect). The dashed line represents the distance between touched points for the corresponding target in the two-tone and grayscale conditions, defining the 'pointing distance' measure. (B) Pointing accuracy for two-tones (colored circles) following grayscale exposure, and grayscales (gray squares), as measured by percentage of touched locations falling within the predefined correct area for each target (pointing accuracy). Small markers show participant means, large markers show age group means, and error bars show bootstrapped 95% confidence intervals. (C) Pointing distance between touched locations on cued two-tone and grayscale conditions of each image in mm (colored circles; all images were displayed at a fixed height of 273.9 mm). Markers and error bars are as in B. (D) Pointing accuracy of previously recognized (white circles) and previously unrecognized (colored circles) two-tones following grayscale exposure. Markers and error bars are as in B. (E) Pointing distance of previously recognized (white circles) and previously unrecognized (colored circles) two-tones following grayscale exposure. Markers and error bars are as in B.

Perceptual reorganization was assessed by measuring two-tone recognition immediately after presenting participants with the grayscale cue. To minimize memory load and make clear that grayscales and two-tones were different versions of the same image, all images were 'morphed' into one another by fading from the grayscale to the overlaid two-tone with position and dimensions maintained. Participants were then asked to touch two predetermined features on each two-tone on the touchscreen (Figure 1A, stage 3). To ensure participants could correctly recognize and point out all targets, they were also asked to locate the corresponding features on the original grayscale images in a control task after the main experiment (Figure 1A, stage 4). Touched coordinates were scored by two methods; first, the percentage of touched points falling within researcher-defined regions gives 'pointing accuracy', and second, the distance between corresponding touched coordinates on two-tone and grayscale images gives 'pointing distance' (see Figure 2A).

There were large age-related changes in cued two-tone recognition, with pointing accuracy and distance both improving substantially with age (Figures 2B and 2C, colored circles). In contrast, for grayscale and Catch images, pointing accuracy was high across all ages (Figure 2B, gray squares; Figure S2B, white circles), so this age difference is unlikely to reflect general changes in task comprehension. However, it is possible that these age differences are driven by factors unrelated to perceptual reorganization, such as adults having recognized more two-tones naively, or children exhibiting greater imprecision or different pointing strategies despite recognizing the content. Therefore, to test for perceptual reorganization while accounting for naive two-tone recognition and pointing skills, we compared pointing performance within subjects for trials in which two-tones were naively recognized before cueing (and therefore likely still recognized after) to trials in which they were not. If grayscale cueing effectively induced perceptual re-organization, pointing performance should be equivalently high across these two trial groups, irrespective of initial recognition. Furthermore, in our mixed-effects modeling analysis (see STAR Methods for details), we accounted for random effects of participants, individual images, ROI size, and unequal numbers of trials.

This analysis showed that a difference in cued pointing performance for naively recognized and unrecognized two-tones was largest for 4- to 5-year-olds and decreased with age (for pointing accuracy: Χ^2^(3) = 13.85, p < 3.11e-3, Figure 2D; for pointing distance: Χ^2^(3) = 16.24, p = 1e-3, Figure 2E), with greater benefits of cueing for older participants on both recognition indices. Wald tests on model coefficients showed that in adults, feature localization was equivalently accurate for naively recognized and unrecognized two-tones (pointing accuracy: *z* = −1.24; p = 0.21; pointing distance: *z* = 0.25; p = 0.8), in line with pervasive perceptual reorganization in the mature system. For pointing accuracy, the grayscale cue was significantly less beneficial for 4- to 5-year-olds than for adults (*z* = 3.22, p = 1.26e-3), marginally less beneficial for 7- to 9-year-olds (*z* = 1.71, p = 0.08), and adult-like for 10- to 12-year-olds (z = 1.46, p = 0.14). For pointing distance, no pairwise age group comparisons reached statistical significance, potentially because this measure was more variable. Image difficulty (number of errors for naming accuracy or pointing accuracy per image) was significantly correlated across naive and cued conditions for 4- to 5-year-olds only (r_pearson’s_ = 0.50, p = 0.02; p > 0.09 for all other groups, see Figure S4 for all correlations). Together, these data reveal that processes supporting adult-like perceptual reorganization, in which two-tone parsing is qualitatively altered when prior knowledge is made available, develop gradually over the first 10 years of life.

### Age differences in cued two-tone parsing strategies

**Figure 3:**
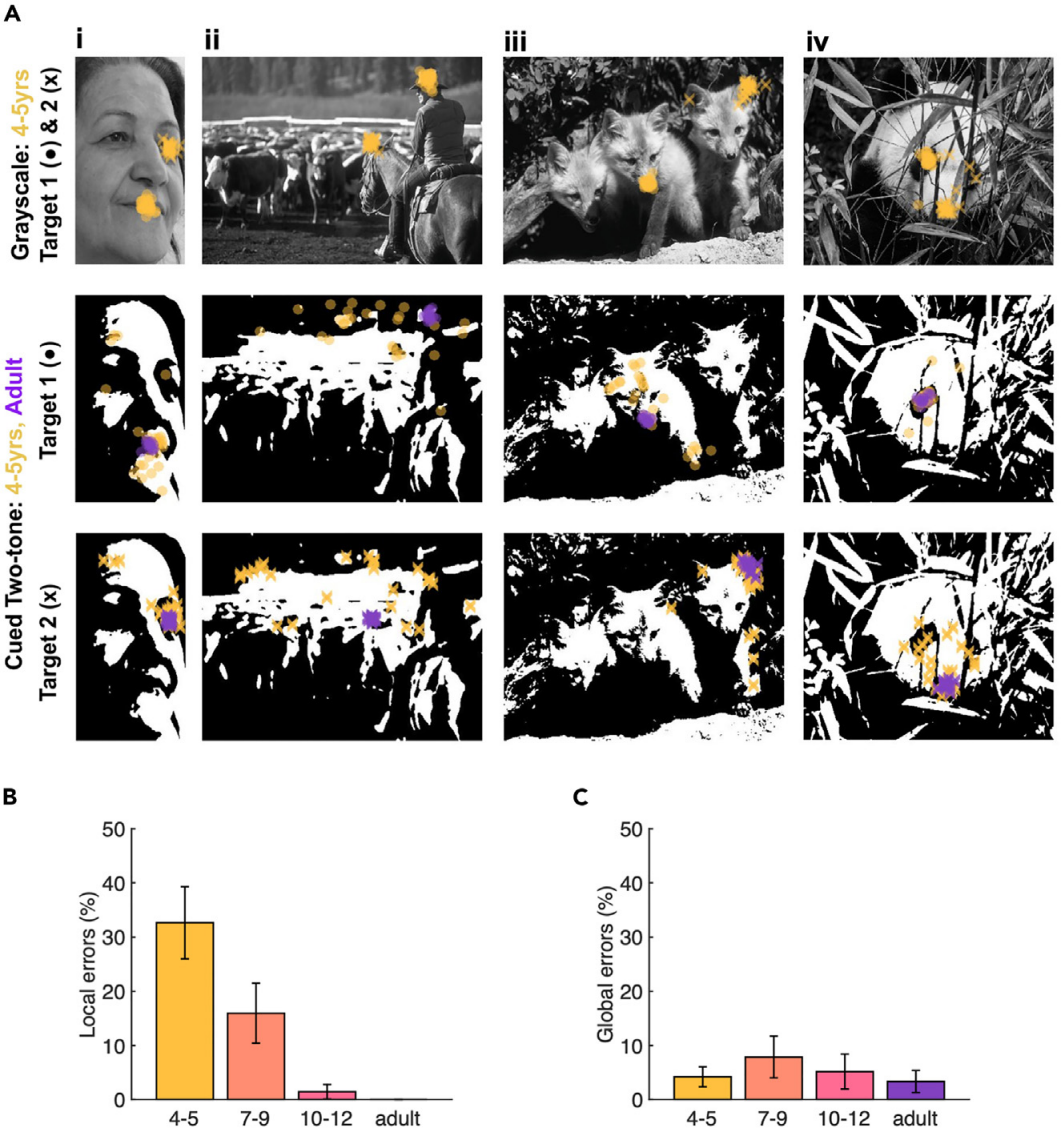
Analysis of cued pointing errors (A) Top row: 4- to 5-year-olds’ grayscale pointing for targets 1 (circles) and 2 (crosses), target prompts were (i) “the lady’s mouth’ (1) and “the lady’s right eye” (2), (ii) “the cowboy’s hat” (1) and “the horse’s left ear” (iii), C “the middle fox’s nose” (1) and “the right fox’s right ear” (2), and (iv) “the panda’s left eye” (1) and “the panda’s nose” (2). Middle and bottom rows show 4- to 5-year-olds’ (yellow) and adults’ (purple) cued two-tone pointing for targets features 1 and 2, respectively. (B) Mean proportion of incorrect responses that constituted 'local errors' (i.e., the selected feature matches the local shape properties of the target, but is positioned incorrectly with regards to global image content) by age group for 8 targets where such locally matching features were present. Error bars show standard error. (C) Mean proportion of incorrect responses that constituted 'global errors' (i.e., the selected feature is incorrect, but is located close to the correct target [within an area three times as large as the correct ROI]). Error bars show standard error.

To investigate age differences in image parsing strategies of cued two-tones, we compared cued pointing responses across ages. We have shown previously that adults typically located both targets on cued two-tones regardless of naive recognition, while 4- to 5-year-olds often failed to locate these targets, and showed poorer performance for naively unrecognized images in particular. Cued pointing patterns reveal that when failing to locate the target feature, 4- to 5-year-olds still seem to display a task-oriented strategy, as illustrated by a high prevalence of local feature-driven errors for this age group (Figure 3). That is, the incorrect selection of two-tone features that canonically resemble the target feature, but whose location does not correspond with the global image content ('local errors'). For example, when asked to locate features of a woman’s face, many children instead located corresponding features of a pareidolic face appearing on the woman’s forehead, despite performing accurately and precisely on the same task in the grayscale condition (Figure 3Ai). Likewise, when asked to point to the cowboy’s hat and horse’s ear, many erroneously located 'hat-like' and 'ear-like' shapes, regardless of their distance from the target features in the just-seen grayscale image (Figure 3Aii). Similarly, when the outlines of target features were obscured following two-tone transformation (e.g., the noses of the fox [Figure 3Aiii, Target 1] and panda [Figure 3Aiv, Target 2]), many children incorrectly selected nearby shapes with more defined outlines. To quantify this pattern of responses across age groups, we measured the proportion of errors displaying a locally feature-driven strategy for eight targets for which incorrect features that matched the target’s shape properties were present in the two-tone (Figure 3B). We compared this to the proportion of errors that fell close to the correct location, displaying a global context-driven strategy (Figure 3C). We found that adults did not commit any local errors for these targets, and for children, the proportion of errors that could be attributed to a local feature-driven strategy decreases dramatically with age. In comparison, the proportion of global errors committed did not differ with age. Together, this suggests that young children who showed little benefit of cueing on image recognition made errors consistent with prioritization of local features, rather than holistic image processing.

### Human development versus computational image recognition models

We next compared human performance on two-tone recognition to that of CNNs to test whether different architectures may correspond to sequential stages in human development. We selected three image recognition models based on their architectural properties and availability; (1) AlexNet^34^ – a shallow feedforward network extensively used in computational neuroscience studies,^3^ (2) CorNet-S, a biologically inspired shallow network that incorporates feedback within late and early-stage processing layers,^35^ and (3) NASNet-Large – a deep feedforward network with a complex branching architecture designed by auto machine learning algorithms optimizing for transferable image classification.^36^ All three models are publicly available and were pre-trained on 1.3 million images from the ImageNet training set into 1,000 different classes.^37^

**Figure 4:**
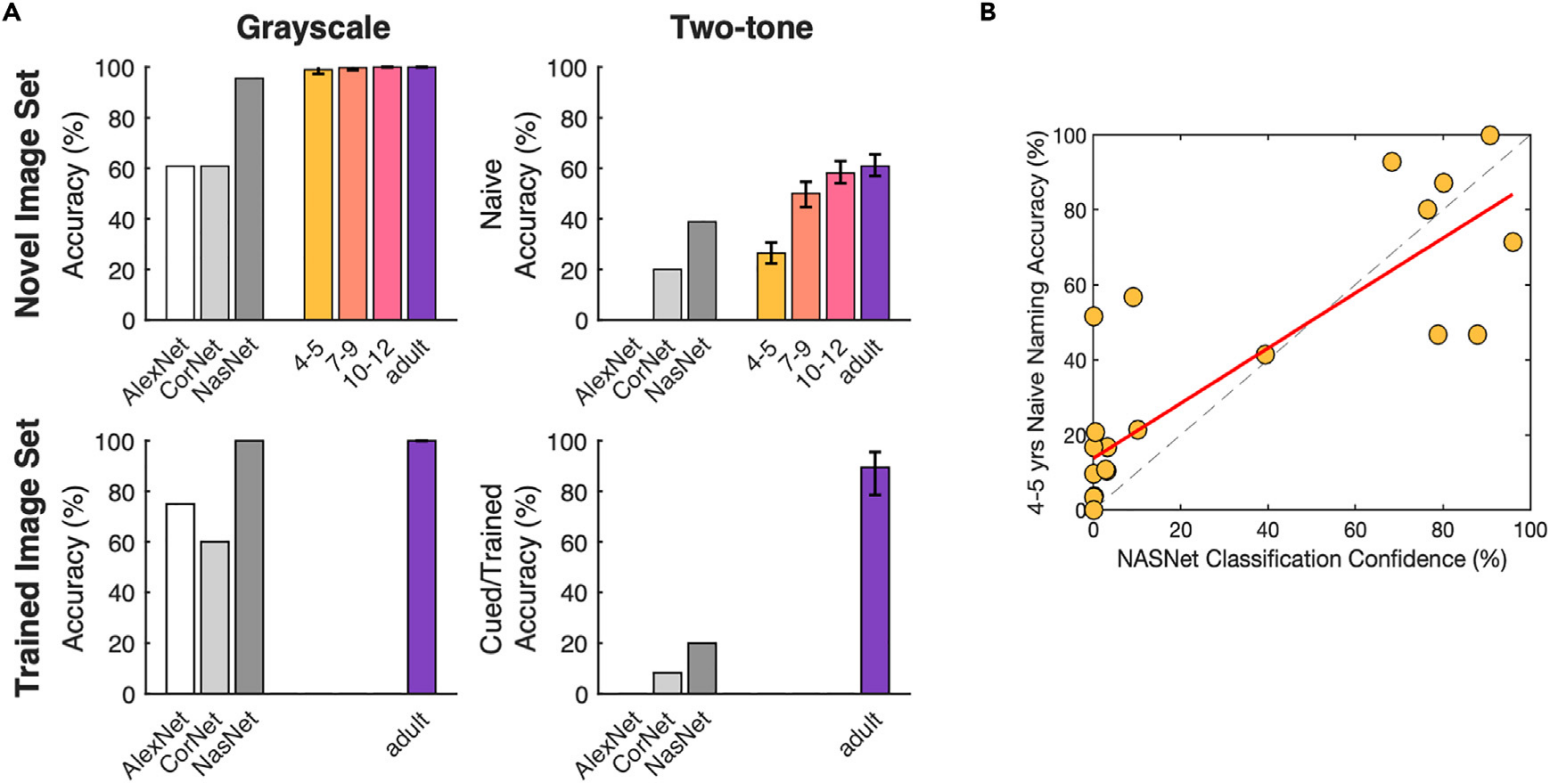
Comparison of CNN performance to human performance (A) Image recognition accuracy of CNNs (AlexNet, CORnet-S, NASNet-large; grayscale bars) and human performance (colored bars, error bars show bootstrapped 95% confidence intervals) for each condition (grayscale and two-tone) of images used in current study (novel Iiage set) and ImageNet images used to train CNN models (trained image set). CNN performance: percent of images correctly labeled; human performance: naive naming accuracy. (B) Imagewise comparison of mean 4- to 5-year-old naive naming accuracy and NASNet performance (measured by probability weighting of the highest-scoring correct label) for Catch and two-tone trials of novel image set. Red line shows linear least-squares line of best fit, dashed line shows x = y.

To assess the possibility of improved two-tone recognition of 'familiar' images compared to novel images (akin to perceptual reorganization) in these CNNs, models were tested on two image sets. First, nineteen of the previously used two-tones that were not included in ImageNet (the dataset used to train all three models) formed the 'novel' image set, and second, twenty comparable two-tones created from ImageNet images formed the 'trained' image set. CNNs with the capacity to benefit from image cueing would be expected to more accurately classify two-tones from 'trained' as opposed to 'novel' image sets. This, however, was not the case for any of the models tested. It is unlikely that this reflects differences in difficulty levels across the two image sets; image pairs across the sets were matched on low-level statistics, piloting in an additional nine adults ensured that two-tone recognition performance was equivalent across the two sets (novel set: 61% naive accuracy, 94% cued accuracy; trained set: 63% naive, 89% cued accuracy), and all CNNs showed comparable performance on the grayscale images of each set (Figure 4A).

We next tested how CNNs performed compared to human participants for naive recognition. We found that NASNet-large was the highest-performing model for both grayscale and two-tone images but nevertheless only reached the performance level of the youngest age group for naive two-tone classification. While both CORnet-S and AlexNet had comparatively lower performance for grayscales than NASNet, for two-tones CORnet did approach naive performance levels of 4- to 5-year-olds while AlexNet failed to classify any two-tones correctly (Figure 4A). Interestingly, NASNet’s classification confidence for two-tones from the 'novel' image set, quantified as the probability weighting of the highest-scoring correct label, correlated across images with human naive naming performance for all ages (Figure 4B, plotted for 4- to 5-year-olds, r_pearson’s_ = 0.84, p = 4.98e-7; see Figure S4 for all correlations). This suggests that despite vastly different computational implementations, there is a common aspect of these two-tone stimuli that poses a challenge to both human vision and CNNs, which adults can overcome when object knowledge is made available.

It is well known that CNNs are vulnerable to image manipulations and have difficulty generalizing to out-of-distribution datasets.^1^ To explore which factors in particular make two-tone images hard to recognize for developing humans and CNNs, we assessed correlations of two-tone recognition with various low-level image features and parameters of the two-tone transformation. Given the proposed need for prior knowledge to resolve edge information for two-tone recognition,^22^^,^^30^ we assessed how two-tone transformation altered various summary statistics of image edge information. Firstly, edge density, as measured by a Sobel edge detector,^38^ was reduced by two-tone transformation as expected (Figure S5B), and larger reductions were associated with poorer naive two-tone recognition in 4- to 5-year-olds and NASNet (r_pearson’s_ > 0.5 and p < 0.01 for both). Next, we considered two biologically principled measures of scene statistics, contrast energy (CE) and spatial coherence (SC), computed via spatial image filters that emulate parvo- and magnocellular operations, respectively^39^ (see Supplementary Materials 5 [S5] for details). Two-tone transformation was found to increase CE but decrease SC, with greater alterations corresponding with poorer two-tone recognition for 4- to 5-year-olds (see Table S5). Together, these correlations may suggest that object recognition in younger children is more sensitive to disruption of edge information in the image than adults, but note that these analyses may be limited by the narrow score ranges in older age groups. However, the extent to which these image statistics correlated with performance did not exceed that of the smoothing levels applied during the two-tone transformation. As expected, increased smoothing was associated with poorer naive two-tone recognition for all ages (r_pearson’s_ < −0.6 and p < 0.001). It was also associated with poorer naive two-tone classification for NASNet and cued two-tone performance for children below the age of ten (see Table S5). These results suggest that the tested measures of scene statistics do not explain what contributes to two-tone difficulty beyond the amount of information that is lost via smoothing.

## Discussion

To test how prior knowledge guides visual object perception between the ages of 4 and 12 years, we sequentially presented ambiguous two-tone images and grayscale cues. In adults, information about a two-tone’s content provided by the original grayscale induces perceptual reorganization, resulting in near-mandatory recognition of previously unrecognizable stimuli. To quantify how this process develops across childhood, we compared the ability to locate features on two-tones that were not recognized naively (i.e., candidate images for perceptual reorganization) to the same measure for two-tones recognized on first viewing. Here, performance on these naively recognized two-tones provides an individual benchmark for feature localization error when image content is perceived. So, if grayscale cueing causes accurate recognition of previously unrecognized two-tones (perceptual reorganization), pointing performance should approach this benchmark. Crucially, this comparison isolates the effects of prior knowledge on two-tone recognition, as age differences in other task-relevant abilities (e.g., pointing precision, biases) should affect both conditions equally.

We first showed that naive two-tone recognition underwent substantial developmental improvement, increasing from 26% to 63% correct between the ages of 4–5 years and adulthood. However, despite this large difference in the effect of visual information loss in two-tones, positive correlations between image difficulties across all age groups showed that the processes hindering naive two-tone recognition remain consistent across age. After seeing the original grayscale, adults and the oldest children could locate target features on previously unrecognized two-tones with similar accuracy as for the two-tones they could recognize naively, clearly demonstrating perceptual reorganization. However, younger children experienced drastically less new recognition of two-tones after seeing the grayscale cue, pointing out substantially fewer image features correctly than their benchmark for naively recognized two-tones. In addition, image difficulty before and after grayscale cueing (i.e., imagewise naive naming and cued pointing accuracy) was correlated for young children, but not for older children and adults. This indicates that only at older ages does prior knowledge induce a qualitative shift in how two-tones are processed. Together, these results reveal that the effective use of prior knowledge in perception poses a major challenge to human development, with improvements occurring gradually between the ages of 4 and 12 years.

It is highly unlikely that this developmental shift in perceptual reorganization reflects age differences in object knowledge, task comprehension, or non-perceptual processes. While the two-tone paradigm intrinsically minimizes potential differences in participants’ object knowledge by providing the relevant information in a task-relevant format, we further controlled for object knowledge differences by only including trials in which grayscale images were correctly named. Task comprehension was also ensured for all participants, both during an initial training phase and throughout the task, via 'easy' two-tone images (Catch images, made by thresholding grayscale images without applying smoothing). High-recognition performance on these trials (Figure S2) demonstrates that all age groups understood, remembered, and followed instructions for both two-tone recognition tasks (naming and pointing) throughout the experiment. In addition, the use of visual stimuli and blurred transitions reduces the demand for working memory, cross-modal integration, and configurement of mental representations. Alternative cueing methods, such as non-sequential paradigms and semantic cues, are sufficient to trigger perceptual reorganization in adults.^25^^,^^40^^,^^41^^,^^42^ While in the present study we prioritized reducing load for cross-modal and working-memory processes known to concurrently develop throughout childhood,^43^^,^^44^^,^^45^ future work should test how these processes affect cue integration across development.

To explore what perceptual strategies younger children employed to parse cued two-tones, we reviewed the patterns of locations that 4- to 5-year-olds selected on these images. This revealed that children often located correct object features if these were clearly outlined, but were prone to large localization errors when object feature boundaries were obscured or disrupted, instead selecting salient features that plausibly matched the target in outline (e.g., when asked to locate a hat, many identified a shape with a hat-like outline, albeit in the wrong location). This error pattern suggests that younger children looked for features by relying on local shape outlines, rather than by identifying and grouping shapes relevant to the object gestalt. Similar strategies have been reported in adults with autism spectrum disorder, who have outperformed non-autistic adults in tasks that favored perception of local rather than global features^46^ (though see Van der Hallen et al.^47^). These individuals also show evidence of reduced effects of prior knowledge of two-tone processing^48^ (though this may be specific to face stimuli^49^^,^^50^), which has been attributed to a relative downweighting of prior knowledge with respect to sensory inputs. Differences in weighting sensory input and prior information have also been implicated in numerous neuropsychiatric disorders.^51^^,^^52^^,^^53^ Indeed, populations that suffer from hallucinations and psychosis-prone individuals experience higher levels of perceptual reorganization of two-tones than healthy adults,^54^^,^^55^^,^^56^ attributed to an upweighting of prior information to compensate for increased noise levels in bottom-up sensory streams.^51^^,^^54^^,^^55^^,^^57^^,^^58^ The severity of symptoms associated with these conditions, and the extensive research ongoing to alleviate them, reemphasizes the importance of characterizing the normal development of top-down processes. Further, many of these disorders are diagnosed in late childhood or adolescence, concurrent with the dramatic changes in top-down weighting shown here. It is therefore an important possibility that the developmental shift we report here could be involved in the onset of neurological pathologies.

Like the youngest children in this study, CNNs have also been shown to prioritize local over global information for object recognition, which too has been linked to higher levels of dependency on feedforward information processing when compared to the adult human visual system.^59^^,^^60^^,^^61^ To assess if this, or other image processing differences of CNNs, corresponds to distinct levels of two-tone recognition, we compared human two-tone recognition to that of CNNs with distinct network architectures. None of the models tested reached naive adult performance, or showed any recognition improvements for 'familiar' two-tones (made from the image set on which models were trained^37^) compared to 'novel' two-tones, despite matching for difficulty. However, NASNet-large,^36^ the deepest model tested and only one to achieve human-like grayscale recognition, reached the two-tone performance level of 4- to 5-year-old children. While CORnet^35^ and AlexNet^34^ showed similarly poor levels of grayscale recognition, CORnet performed better on two-tones for both trained and novel image sets. These results suggest that training with the original image used to make a two-tone may not be sufficient to trigger an analog of perceptual reorganization in these CNNs. Due to the common training set of these models, the differences in naive two-tone recognition seen here are likely driven by architectural features. Namely, increased model depth may have afforded NASNet-large improved performance for both grayscale and two-tone images compared to the other shallower models, while the addition of recurrency may drive CORNet’s advantage for two-tones over AlexNet, which is both shallow and completely feedforward. It is important to note, however, that it is possible for purely feedforward models to achieve equivalently 'recurrent' computations as CNNs with feedback connections,^62^ which may explain why NASNet-large was able to outperform a more biologically inspired model. Of course, CNNs differ greatly from the human visual system; in addition to the local biases discussed previously, Geirhos and colleagues^61^ found that CNNs prefer images that are colored or high contrast. These biases may have inhibited general recognition of our stimuli set, none of which included color information, but less so for Catch images, which were high contrast but unsmoothed (Figure S2C). Interestingly, despite large mechanistic differences, NASNet and human participants showed correlated imagewise performance on naive two-tones, revealing commonalities in the challenges these images pose for machine and human visual systems, especially those still developing.

To explore what these challenges may be, we tested how low-level spatial image properties correlated with two-tone recognition performance in developing humans and CNNs. As expected, the smoothing and thresholding levels used in the two-tone transformation process negatively correlated with naive recognition across ages in humans, and for NASNet. This is unsurprising, as higher smoothing levels result in a greater loss of information, and higher thresholds effectively increase image shadows. As these image manipulations can also be expected to interact in a non-linear or unpredictable manner, we used physiologically plausible models of image statistics extraction to quantify the effects of two-tone transformation on low-level spatial image properties (i.e., edge energy, contrast energy, and spatial coherence) computed at early visual processing stages.^39^ While the effects of smoothing and binarizing images clearly shifted the low-level spatial properties, we did not find that these statistics correlated better with performance than smoothing levels alone. Nonetheless, as only 24 images were included to ensure a child-friendly task duration, this study was not optimized to model the effect of image properties on perception. Studies with larger image sets that can systematically compare the effects of different image features will therefore be necessary to assess the bottom-up contributions to two-tone perception across development.

Another factor that may contribute to the development of two-tone perception is the efficiency of spatial integration mechanisms across the cortical hierarchy. Two-tone perception requires both the detection and segmentation of object contours from irrelevant contours (i.e., from cast shadows, occluders, and background objects) and the integration of these relevant contours to form the figural percept.^22^^,^^63^ There is evidence that contour integration develops substantially in childhood: Kovács et al.^64^ showed that the ability to detect contours consisting of collinear-oriented elements amid misaligned distractor elements improves substantially between 4 and 12 years of age. Unlike adults, children were less tolerant to distractors when there were larger spacings between the contour elements, suggesting a limitation on spatial integration distance rather than a reduced ability to detect signal in noise. Similarly, the perception of illusory ''Kanizsa'' shapes, in which shape corners are visible but the contours connecting these corners are not, has been shown to develop gradually over the first decade of life.^65^ Though these stimuli are simpler than two-tones, the perception of Kanizsa shapes has also been shown to involve hierarchically organized feedback processing.^66^^,^^67^ More complex images and those with less clearly defined object boundaries have been shown to require higher levels of recurrent processing in order to extract the image content^68^^,^^69^; a process that can be silenced by disrupting higher-order visual areas.^69^^,^^70^ An increase in top-down signal integration with sensory inputs across childhood may therefore provide a common explanation for the prolonged development of these perceptual tasks.

While we carefully matched visual object knowledge of the stimulistimuli across age groups, it is possible that the primed object representation was still less abstract, or 'invariant' to image distortions, in children (and CNNs), thus offering a less robust template for parsing the two-tone. Indeed, in a cross-cultural study, adults from an isolated tribe with little-to-no experience with pictorial representations benefitted less from grayscale cueing than Western adults.^71^ We made every effort to make clear that two-tones and grayscales were two versions of the same photos by blurring corresponding images into each other. It is however possible that benefiting from grayscale cueing requires an understanding of dual representations, which children have been shown to lack.^72^ It thus remains to be tested whether children (or computer vision models) would experience higher levels of perceptual reorganization after extensive training with the depicted objects in varying orientations, sizes, and depictions, and/or the two-tone form.

In sum, we show that throughout most of childhood the influenceinfluence of prior knowledge on two-tone recognition is drastically lower thanlower than in the mature visual system. When compared to adults, young children may focus more heavily on local image features, characteristic of bottom-up processing streams. We found evidence of these strategies in young children, whose image recognition also correlated with CNN performance – which has previously been shown to exhibit local biases in image processing. For both these visual systems, the development of prior-knowledge-driven perception, a central aspect of adult human vision, could depend on the formation of more invariant or abstract object representations or increased connections across the processing hierarchy that enable informed integration of incoming spatial features.

### Limitations of the study

The current findings of this study demonstrate a late development of perceptual reorganization of two-tone images. However, whether this development is driven by the acquisition of more invariant object representations or enhanced integration of top-down information cannot be inferred by this study. Furthermore, the extent to which this late development is generalizable across different types of visual stimuli, or indeed other sensory modalities, remains unclear. Thirdly, as personal details of participants including gender were not stored, analyses into the effect of these characteristics on levels of perceptual reorganization are not possible within the current study. We aim to address these points in further behavioral and neuroimaging studies on the topic. In addition, our comparison of human performance with that of CNNs reveals that these models of computer vision were not able to overcome the information loss in these stimuli, despite varying model architectures. However, as multiple aspects of these architectures varied between models, we cannot draw conclusions about the extent to which certain features accounted for accurate classification of these stimuli, and as all models were pre-trained, neither are we able to assess the effect of image cueing on two-tone classification.

## STAR★Methods

### Key resources table

**Table:** 

REAGENT or RESOURCE	SOURCE	IDENTIFIER
**Deposited data**		

Data and codes for all the figures	This paper	www.github.com/orgs/ChildVisionLab/projects/2

**Software and algorithms**		

Matlab R2015b	Mathworks^73^	RRID: SCR_001622
Matlab Deep Learning Toolbox	Mathworks^38^	RRID: SCR_024747
Psychophysics Toolbox	Brainard^74^	RRID: SCR_002881
Python	Python Software Foundation	RRID: SCR_008394
R package: lme4	Bates^75^	RRID: SCR_015654

### Resource availability

#### Lead contact

Further information and requests for code should be directed to and will be fulfilled by the lead contact, Georgia Milne (georgia.milne@ucl.ac.uk).

#### Materials availability

This study did not generate new materials.

#### Data and code availability

• All data reported in this paper will be shared by the lead contact upon request. All original code has been deposited at www.github.com/orgs/ChildVisionLab/projects/2 and is publicly available as of the date of publication.
• DOIs are listed in the key resources table.
• Any additional information required to reanalyze the data reported in this paper is available from the lead contact upon request.

### Experimental model and study participant details

Behavioral participants were 74 children aged 3 - 13 years and 14 adults: 31 4- to 5-year-olds (mean age = 4.8, SD = 0.5 years, range = 3.1 - 5.7 years), 23 7- to 9-year-olds (mean age = 8.5, SD = 0.9 years, range = 6.8 - 9.7 years), 18 10- to 12-year-olds (mean age = 11.0, SD = 0.8 years, range = 10.2 - 13.7) and 13 adults (mean age = 22.2, SD = 2.2 years, range = 19.3 - 25.8 years). Age groups and sample sizes were defined based on effect sizes found in previous studies.^13^^,^^33^ Three 4- to 5-year-olds, one 7- to 9-year-old and one adult participant were excluded due to not completing the experiment or not complying with the task. An additional nine adult participants (mean age = 29.7, SD = 5.2 years, range = 23.7 38.9 years) completed piloting of the second stimuli set (‘trained’ set, see convolutional neural networks*).*

All participants had normal or corrected-to-normal vision, no reported history of eye disease, developmental or neurological disorder, or premature birth. Adults were recruited through the UCL Psychology Subject Pool (“SONA”) and received £10.50/hour compensation. Children were recruited through the UCL Child Vision Lab volunteer database, and received certificates, small toys, and transportation costs. Informed written consent was obtained from all adults and parents, and children gave verbal assent. Personal details including participant sex, gender and ethnicity were not stored, and therefore analyzes of these data are not reported. The research was carried out in accordance with the tenets of the Declaration of Helsinki, and was approved by the UCL Ethics Committee (#1690/005).

### Method details

#### Apparatus

Stimuli were presented on a 22” touchscreen monitor (Iiyama ProLite T2252MTS 22”, 1900x1080 pixel resolution) driven by a MacbookPro, running Matlab R2015b^73^ with the Psychophysics Toolbox.^74^

#### Behavioral stimuli

Stimuli consisted of 20 two-tones created by processing grayscale photographs of objects, animals and faces in Matlab R2015b^73^; images were first smoothed with a Gaussian filter and then thresholded to binarise pixel luminance to black or white to create images with obscured edges. Smoothing levels and binarisation thresholds varied per image. Images were selected from an original set of 41 stimuli following piloting with 13 adults and 16 children (4-10 years) for their effectiveness as two-tones, feasibility for testing young children, and to cover a range of difficulties for two-tone recognition. Selection criteria included high recognition accuracy (>95%) of grayscale versions at all ages, low recognition accuracy of naively viewed two-tones, and comparatively high recognition accuracy of cued two-tones. An additional 7 two-tones were generated without smoothing, creating easily recognisable two-tones, of which 3 were used in practice trials and 4 were used in ‘Catch’ trials, included to promote motivation, and obtain an index of attentiveness and task comprehension. All images were resized to a fixed height of 273.9 mm (680 pixels, ∼30 dva) on a mid-grey background. Text task prompts were displayed above the image and read aloud by the researcher.

#### Procedure

Participants sat ∼30 cm in front of the touchscreen monitor that was in an upright position. Ambient lighting was controlled to maintain constant dim lighting. The experimenter was present throughout the procedure, and caregivers were sometimes present but did not engage with the procedure. Following task instruction, participants completed a training task in which a grayscale image was displayed and transformed gradually into a two-tone image that was unsmoothed and easy to recognise for all ages. To confirm that participants had understood that image content was maintained across the grayscale and two-tone, they were asked to point out corresponding features between the original grayscale image and the two-tone image when displayed side by side. Following this, participants completed 3 practice trials with unsmoothed two-tones. This was followed by 20 experimental trials with two-tones of varying difficulty, presented in a randomised order and interspersed with easy ‘Catch’ trials every 5th trial, following which prize tokens were awarded to maintain motivation and a short break was allowed. Motivational, but uninformative, feedback was given after each trial to all participants.

Trials consisted of 3 sequential stages (Figure 1A). First, participants were permitted to free-view the uncued two-tone for an unlimited time, and asked to identify its content (stage 1). Once the participant answered, or was unwilling to guess after being prompted to, this two-tone was overlaid with its original grayscale image, preserving image size and position, and participants were asked to identify this image (stage 2). To demonstrate how the grayscale image is ‘transformed’ to the previously seen two-tone version, these images were then ‘morphed’ into the previously seen two-tone version via sequentially thresholding the grayscale with a decreasing number of channels represented (5 steps in total from 255 to 2 channels, with smoothing applied on the final step; 3-second duration in total). Participants were asked to confirm recognition of the two-tone by pointing out two defining features on the image (stage 3). After completing all 20 experimental trials and 4 Catch trials, participants were then asked to locate the ROIs they had pointed out on the two-tone on each of the 24 corresponding grayscales. Verbal responses and screen coordinates of touched image locations were recorded for each trial. All task instructions were displayed as text above the stimulus image and also given verbally by the experimenter. The task duration was approximately 15 minutes including short breaks.

#### Scoring of behavioral performance

To quantify image recognition, we measured ‘naive naming accuracy’, ‘cued pointing accuracy’, and ‘cued pointing distance’. *Naive naming accuracy*: Image names were scored as correct if the content was correctly identified at the basic category level^76^ - superordinate categories names (e.g., naming a ‘cow’ an ‘animal’) were scored as incorrect. Similar basic level or subordinate categories were accepted (e.g., a ‘tiger’ named as ‘cat’, or ‘scissors’ named as ‘shears’) as long as there was consistency in naming across the two-tone and grayscale (for all answers and coding scheme details per image, see Table S1). Trials were excluded from Cued Pointing analyzes if both targets were not attempted for both grayscale and two-tone conditions, and if participants were unable to accurately name the image content or locate each target in the grayscale condition. *Cued pointing accuracy*: pointed out feature location was scored as correct if the touched coordinates fell within a researcher-defined region of interest (ROI) demarcating the location of each target on both the two-tone and grayscale images. *Cued pointing distance*: the distance between each touched location for grayscale targets and the corresponding two-tone targets in millimetres. For the analysis of local errors, we considered eight targets for which incorrect features matching the target’s shape properties were present in the two-tone. Of the total errors made for these targets, the proportion whose touched coordinates fell within an ROI demarcating the incorrect shape-matching feature was counted for each age group. ROIs were equal in area to those used for the Pointing Accuracy measure described above.

#### Convolutional neural networks

Pretrained AlexNet and NASNet-large models were acquired from and run with Matlab R2020a’s Deep Learning Toolbox.^38^ CORnet models (RT, Z and S) were acquired and run with the Python toolbox THINGSvision.^77^ All models were run on a 2015 MacBook Pro. Due to poorer grayscale performance, CORnet-RT and CORnet-Z were not included in the results. All models were pre-trained to classify images into 1000 classes on the ImageNet dataset.^37^ Following the same coding scheme for human participants described above, CNN Classification Accuracy was determined separately for grayscale and two-tone images of ‘trained’ and ‘novel’ image sets (see CNN stimuli) by whether the first-choice (highest weighted) classification was correct (with at least a basic-level match). Classification Probability was measured as the probability weighting of the highest weighted label scored as correct as per the coding scheme within the top 100 predictions.

#### CNN stimuli

Thirty-five images were selected from ImageNet to create additional grayscales and two-tones, chosen due to similarities with the behavioral stimuli set and suitability for two-tone transformation. Resulting grayscales and two-tones were dynamically cropped to a 680∗680-pixel square to minimise loss of image content. Image piloting was carried out in eight additional adult participants using an adapted version of the behavioral task described above: Images were shown in 7 blocks of 5 images, where 5 naive two-tone trials were followed by 5 corresponding grayscale trials and finally 5 cued two-tone trials, with image order randomised within conditions. Recognition was determined via Naming Accuracy (as above) for all three conditions, with an additional perceptual check (‘Which way is the animal/object facing?’) asked as confirmation for scoring cued trials correct. Following piloting, 20 images were selected to match the behavioral stimuli on smoothing, thresholding and adult performance levels. Of the 20 behavioral stimuli described above, 19 that were not found within the ImageNet dataset (one grayscale - two-tone pair excluded; behavioral image 6, see Table S1) comprised the ‘novel’ image set. Images of both sets were resized and triplicated across RGB channels to match model input sizes (AlexNet: 227∗227∗3 pixels, NASNet: 331∗331∗3 pixels, CORnet-S: 224∗224∗3 pixels).

### Quantification and statistical analysis

To analyze responses, we used a mixed-effects modelling approach, as this allowed us to account for different numbers of trials per condition and age groups, and random effects of individual participants and images. We used generalised linear models estimated using the lme4 library in R.^75^ To compare naming we used multilevel logistic regression models with crossed random intercepts for participants and images. To compare pointing accuracy, we used multilevel logistic regression models with crossed random intercepts for participants, images, and ROI area in pixels. For distance measures, we used a multilevel generalised linear model with a log link function (as pointing error is strictly positive, distance data were log-transformed to ensure normality), with crossed random intercepts for participants, images, and ROI area. To test for age trends, we tested each model against a reduced model without the age effect of interest, using a likelihood ratio test (data and analysis code available on request). We performed Wald tests on the coefficients for pair-wise age group comparisons. Statistical details of the above tests are reported in the Results text and Supplementary Materials, with statistical significance defined as *p* < 0.05.

## References

[1] Geirhos R., Temme C.R.M., Rauber J., Schütt H.H., Bethge M., Wichmann F.A. (2020). Generalisation in humans and deep neural networks. arXiv.

[2] Huber L.S., Geirhos R., Wichmann F.A. (2023). The developmental trajectory of object recognition robustness: children are like small adults but unlike big deep neural networks. J. Vis..

[3] Lindsay G.W. (2021). Convolutional Neural Networks as a Model of the Visual System: Past, Present, and Future. J. Cognit. Neurosci..

[4] Pei Y., Huang Y., Zou Q., Zhang X., Wang S. (2021). Effects of Image Degradation and Degradation Removal to CNN-Based Image Classification. IEEE Trans. Pattern Anal. Mach. Intell..

[5] Slater A., Morison V. (1985). Shape constancy and slant perception at birth. Perception.

[6] Bomba P.C., Siqueland E.R. (1983). The nature and structure of infant form categories. J. Exp. Child Psychol..

[7] Quinn P.C. (2002). Category Representation in Young Infants. Curr. Dir. Psychol. Sci..

[8] Clerkin E.M., Hart E., Rehg J.M., Yu C., Smith L.B. (2017). Real-world visual statistics and infants' first-learned object names. Philos. Trans. R. Soc. Lond. B Biol. Sci..

[9] Smith L.B. (2009). From Fragments to Geometric Shape: Changes in Visual Object Recognition Between 18 and 24 Months. Curr. Dir. Psychol. Sci..

[10] Althaus N., Westermann G. (2016). Labels constructively shape object categories in 10-month-old infants. J. Exp. Child Psychol..

[11] Arias-Trejo N., Plunkett K. (2009). Lexical-semantic priming effects during infancy. Philos. Trans. R. Soc. Lond. B Biol. Sci..

[12] Bova S.M., Fazzi E., Giovenzana A., Montomoli C., Signorini S.G., Zoppello M., Lanzi G. (2007). The Development of Visual Object Recognition in School-Age Children. Dev. Neuropsychol..

[13] Dekker T., Mareschal D., Sereno M.I., Johnson M.H. (2011). Dorsal and ventral stream activation and object recognition performance in school-age children. Neuroimage.

[14] Nishimura M., Scherf S., Behrmann M. (2009). Development of object recognition in humans. F1000 Biol. Rep..

[15] Bar M. (2004). Visual objects in context. Nat. Rev. Neurosci..

[16] Friston K. (2010). The free-energy principle: a unified brain theory?. Nat. Rev. Neurosci..

[17] Kersten D., Mamassian P., Yuille A. (2004). Object perception as Bayesian inference. Annu. Rev. Psychol..

[18] Seijdel N., Loke J., Van de Klundert R., Van der Meer M., Quispel E., Van Gaal S., de Haan E.H.F., Scholte H.S. (2021). On the necessity of recurrent processing during object recognition: It depends on the need for scene segmentation. J. Neurosci..

[19] Baum G.L., Cui Z., Roalf D.R., Ciric R., Betzel R.F., Larsen B., Cieslak M., Cook P.A., Xia C.H., Moore T.M. (2020). Development of structure–function coupling in human brain networks during youth. Proc. Natl. Acad. Sci. USA.

[20] Fair D.A., Dosenbach N.U.F., Church J.A., Cohen A.L., Brahmbhatt S., Miezin F.M., Barch D.M., Raichle M.E., Petersen S.E., Schlaggar B.L. (2007). Development of distinct control networks through segregation and integration. Proc. Natl. Acad. Sci. USA.

[21] Mooney C.M. (1957). Age in the development of closure ability in children. Can. J. Psychol..

[22] Moore C., Cavanagh P. (1998). Recovery of 3D volume from 2-tone images of novel objects. Cognition.

[23] Bona S., Cattaneo Z., Silvanto J. (2016). Investigating the Causal Role of rOFA in Holistic Detection of Mooney Faces and Objects: An fMRI-guided TMS Study. Brain Stimul..

[24] Flounders M.W., González-García C., Hardstone R., He B.J. (2019). Neural dynamics of visual ambiguity resolution by perceptual prior. Elife.

[25] González-García C., Flounders M.W., Chang R., Baria A.T., He B.J. (2018). Content-specific activity in frontoparietal and default-mode networks during prior-guided visual perception. Elife.

[26] Hardstone R., Zhu M., Flinker A., Melloni L., Devore S., Friedman D., Dugan P., Doyle W.K., Devinsky O., He B.J. (2021). Long-term priors influence visual perception through recruitment of long-range feedback. Nat. Commun..

[27] Hsieh P.-J., Vul E., Kanwisher N. (2010). Recognition Alters the Spatial Pattern of fMRI Activation in Early Retinotopic Cortex. J. Neurophysiol..

[28] Imamoglu F., Kahnt T., Koch C., Haynes J.-D. (2012). Changes in functional connectivity support conscious object recognition. Neuroimage.

[29] van Loon A.M., Fahrenfort J.J., van der Velde B., Lirk P.B., Vulink N.C.C., Hollmann M.W., Scholte H.S., Lamme V.A.F. (2016). NMDA Receptor Antagonist Ketamine Distorts Object Recognition by Reducing Feedback to Early Visual Cortex. Cerebr. Cortex.

[30] Teufel C., Dakin S.C., Fletcher P.C. (2018). Prior object-knowledge sharpens properties of early visual feature-detectors. Sci. Rep..

[31] Lee T.S., Mumford D. (2003). Hierarchical Bayesian inference in the visual cortex. J. Opt. Soc. Am..

[32] Kovács I., Eisenberg M. (2005).

[33] Yoon J.M.D., Winawer J., Witthoft N., Markman E.M. (2007). Presented at the 2007 IEEE 6th International Conference on Development and Learning, IEEE, London, UK.

[34] Krizhevsky A., Sutskever I., Hinton G.E. (2017). ImageNet classification with deep convolutional neural networks. Commun. ACM.

[35] Kubilius J., Schrimpf M., Kar K., Rajalingham R., Hong H., Majaj N., Issa E., Bashivan P., Prescott-Roy J., Schmidt K. (2019). Brain-Like Object Recognition with High-Performing Shallow Recurrent ANNs 12. arXiv.

[36] Zoph B., Vasudevan V., Shlens J., Le Q.V. (2018). Presented at the 2018 IEEE/CVF Conference on Computer Vision and Pattern Recognition (CVPR).

[37] Deng J., Dong W., Socher R., Li L.-J., Li K., Fei-Fei L. (2009). Presented at the 2009 IEEE Computer Society Conference on Computer Vision and Pattern Recognition Workshops (CVPR Workshops), IEEE, Miami, FL.

[38] Mathworks (2020).

[39] Groen I.I.A., Ghebreab S., Prins H., Lamme V.A.F., Scholte H.S. (2013). From Image Statistics to Scene Gist: Evoked Neural Activity Reveals Transition from Low-Level Natural Image Structure to Scene Category. J. Neurosci..

[40] Ludmer R., Dudai Y., Rubin N. (2011). Uncovering Camouflage: Amygdala Activation Predicts Long-Term Memory of Induced Perceptual Insight. Neuron.

[41] Nordhjem B., Kurman Petrozzelli C.I., Gravel N., Renken R.J., Cornelissen F.W. (2015). Eyes on emergence: Fast detection yet slow recognition of emerging images. J. Vis..

[42] Samaha J., Boutonnet B., Postle B.R., Lupyan G. (2018). Effects of meaningfulness on perception: Alpha-band oscillations carry perceptual expectations and influence early visual responses. Sci. Rep..

[43] Buss A.T., Ross-Sheehy S., Reynolds G.D. (2018). Visual working memory in early development: a developmental cognitive neuroscience perspective. J. Neurophysiol..

[44] Jüttner M., Müller A., Rentschler I. (2006). A developmental dissociation of view-dependent and view-invariant object recognition in adolescence. Behav. Brain Res..

[45] Swanson H.L. (2017). Verbal and visual-spatial working memory: What develops over a life span?. Dev. Psychol..

[46] Happé F.G.E., Booth R.D.L. (2008). The Power of the Positive: Revisiting Weak Coherence in Autism Spectrum Disorders. Q. J. Exp. Psychol..

[47] Van der Hallen R., Evers K., Brewaeys K., Van den Noortgate W., Wagemans J. (2015). Global processing takes time: A meta-analysis on local–global visual processing in ASD. Psychol. Bull..

[48] Król M., Król M. (2019). The world as we know it and the world as it is: Eye-movement patterns reveal decreased use of prior knowledge in individuals with autism. Autism Res..

[49] Loth E., Gómez J.C., Happé F. (2010). When seeing depends on knowing: Adults with Autism Spectrum Conditions show diminished top-down processes in the visual perception of degraded faces but not degraded objects. Neuropsychologia.

[50] Van de Cruys S., Vanmarcke S., Van de Put I., Wagemans J. (2018). The Use of Prior Knowledge for Perceptual Inference Is Preserved in ASD. Clin. Psychol. Sci..

[51] Fletcher P.C., Frith C.D. (2009). Perceiving is believing: a Bayesian approach to explaining the positive symptoms of schizophrenia. Nat. Rev. Neurosci..

[52] Park S., Zikopoulos B., Yazdanbakhsh A. (2022). Visual illusion susceptibility in autism: A neural model. Eur. J. Neurosci..

[53] Shanmugan S., Wolf D.H., Calkins M.E., Moore T.M., Ruparel K., Hopson R.D., Vandekar S.N., Roalf D.R., Elliott M.A., Jackson C. (2016). Common and Dissociable Mechanisms of Executive System Dysfunction Across Psychiatric Disorders in Youth. Aust. J. Pharm..

[54] Davies D.J., Teufel C., Fletcher P.C. (2018). Anomalous Perceptions and Beliefs Are Associated With Shifts Toward Different Types of Prior Knowledge in Perceptual Inference. Schizophr. Bull..

[55] Teufel C., Subramaniam N., Dobler V., Perez J., Finnemann J., Mehta P.R., Goodyer I.M., Fletcher P.C. (2015). Shift toward prior knowledge confers a perceptual advantage in early psychosis and psychosis-prone healthy individuals. Proc. Natl. Acad. Sci. USA.

[56] Zarkali A., Adams R.A., Psarras S., Leyland L.-A., Rees G., Weil R.S. (2019). Increased weighting on prior knowledge in Lewy body-associated visual hallucinations. Brain Commun..

[57] Kapur S. (2003). Psychosis as a State of Aberrant Salience: A Framework Linking Biology, Phenomenology, and Pharmacology in Schizophrenia. Aust. J. Pharm..

[58] Rivolta D., Castellanos N.P., Stawowsky C., Helbling S., Wibral M., Grützner C., Koethe D., Birkner K., Kranaster L., Enning F. (2014). Source-Reconstruction of Event-Related Fields Reveals Hyperfunction and Hypofunction of Cortical Circuits in Antipsychotic-Naive, First-Episode Schizophrenia Patients during Mooney Face Processing. J. Neurosci..

[59] Baker N., Lu H., Erlikhman G., Kellman P.J. (2018). Deep convolutional networks do not classify based on global object shape. PLoS Comput. Biol..

[60] Brendel W., Bethge M. (2019). Approximating CNNs with Bag-of-local-Features models works surprisingly well on ImageNet. arXiv.

[61] Geirhos R., Rubisch P., Michaelis C., Bethge M., Wichmann F.A., Brendel W. (2019). ImageNet-trained CNNs are biased towards texture; increasing shape bias improves accuracy and robustness. arXiv.

[62] van Bergen R.S., Kriegeskorte N. (2020). Going in circles is the way forward: The role of recurrence in visual inference. Curr. Opin. Neurobiol..

[63] Poltoratski S., Tong F. (2020). Resolving the Spatial Profile of Figure Enhancement in Human V1 through Population Receptive Field Modeling. J. Neurosci..

[64] Kovács I., Kozma P., Fehér A., Benedek G. (1999). Late maturation of visual spatial integration in humans. Proc. Natl. Acad. Sci. USA.

[65] Nayar K., Franchak J., Adolph K., Kiorpes L. (2015). From local to global processing: The development of illusory contour perception. J. Exp. Child Psychol..

[66] Kok P., Bains L.J., van Mourik T., Norris D.G., de Lange F.P. (2016). Selective Activation of the Deep Layers of the Human Primary Visual Cortex by Top-Down Feedback. Curr. Biol..

[67] Wokke M.E., Vandenbroucke A.R.E., Scholte H.S., Lamme V.A.F. (2013). Confuse Your Illusion: Feedback to Early Visual Cortex Contributes to Perceptual Completion. Psychol. Sci..

[68] Groen I.I.A., Jahfari S., Seijdel N., Ghebreab S., Lamme V.A.F., Scholte H.S. (2018). Scene complexity modulates degree of feedback activity during object detection in natural scenes. PLoS Comput. Biol..

[69] Kirchberger L., Mukherjee S., Schnabel U.H., van Beest E.H., Barsegyan A., Levelt C.N., Heimel J.A., Lorteije J.A.M., van der Togt C., Self M.W., Roelfsema P.R. (2021). The essential role of recurrent processing for figure-ground perception in mice. Sci. Adv..

[70] Wokke M.E., Sligte I.G., Steven Scholte H., Lamme V.A.F. (2012). Two critical periods in early visual cortex during figure–ground segregation. Brain Behav..

[71] Yoon J.M.D., Witthoft N., Winawer J., Frank M.C., Everett D.L., Gibson E. (2014). Cultural Differences in Perceptual Reorganization in US and Pirahã Adults. PLoS One.

[72] DeLoache J.S., Miller K.F., Rosengren K.S. (1997). The Credible Shrinking Room: Very Young Children’s Performance With Symbolic and Nonsymbolic Relations. Psychol. Sci..

[73] MathWorks (2015). https://www.mathworks.com.

[74] Brainard D.H. (1997). The Psychophysics Toolbox. Spatial Vis..

[75] Bates D., Mächler M., Bolker B., Walker S. (2015). Fitting Linear Mixed-Effects Models Using lme4. J. Stat. Software.

[76] Rosch E., Mervis C.B., Gray W.D., Johnson D.M., Boyes-Braem P. (1976). Basic objects in natural categories. Cognit. Psychol..

[77] Muttenthaler L., Hebart M.,N. (2021). THINGSvision: a Python toolbox for streamlining the extraction of activations from deep neural networks. Front. Neuroinf..

