# Supplementary material for: Perceptual reorganization from prior knowledge emerges late in childhood
Source: iScience. 2024 Jan 4;27(2):108787. doi: 10.1016/j.isci.2024.108787 (PMC10831247; doi:10.1016/j.isci.2024.108787)
Supplement: Document S1. Figures S2–S5 and Tables S1 and S5 [file mmc1.pdf]

iScience, Volume *27*

Supplemental information

Perceptual reorganization from prior knowledge emerges late in childhood

Georgia A. Milne, Matteo Lisi, Aisha McLean, Rosie Zheng, Iris I.A. Groen, and Tessa M. Dekker

Supplementary Materials

Table S1: Naming Accuracy Coding Scheme, *Related to STAR Methods*

Table shows accepted answers for Catch trials (rows 1-4), Behavioural Stimuli (rows 5-24) and 'Trained' Stimuli (rows 25 - 45). Asterisk denotes answers which were only accepted if consistent with corresponding greyscale answers (for behavioural trials) or if no other more applicable classification was available (for CNNs).

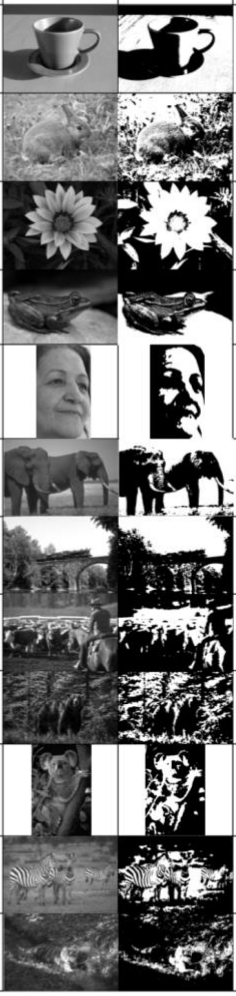

	Greyscale	Two-tone	Accepted Answers
Catch 1			Cup, Mug, Coffee Mug, Teacup, Glass*, Teapot*, Coffeepot*
Catch 2			Rabbit, Type of rabbit, Bunny, Hare Squirrel*, Badger*
Catch 3			Flower, Sunflower, Daisy
Catch 4			Frog, Type of frog, Toad, Lizard*, Type of lizard*
Behav. 1			Woman, Lady, Girl, Face, Person, Human, Man*, Eyes*, Bob Marley*
Behav. 2			Elephant, Elephants, Tusker
Behav. 3			Train, Bridge, Type of bridge
Behav. 4			Cowboy, Horseman, Man/Person on horse, Horse, Person*, Man*, Cow*, Cowboy hat*
Behav. 5			Bear, Brown bear, Polar Bear*, Panda*, Bears*
Behav. 6			Koala, Sloth*, Monkey*, Bear*, Squirrel*
Behav. 7			Zebra, Zebras
Behav. 8			Tiger, Lion*, Cat*, Wildcat*

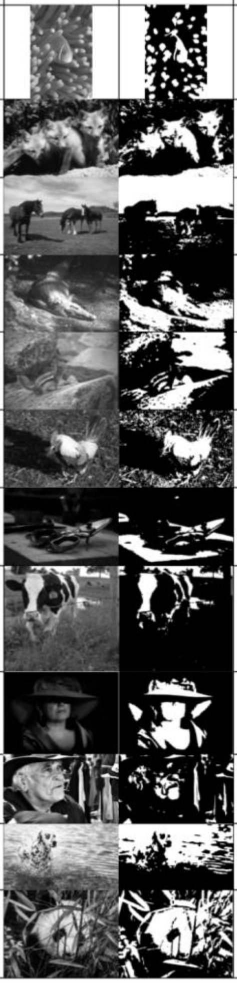

	Greyscale	Two-tone	Accepted Answers
Behav. 9			Fish, Nemo, Anemone fish, Coral reef
Behav. 10			Fox, Foxes, Fox cubs, Type of fox, Cats*, Kittens*, Dogs*, Pups*, Wolves*, Cubs*, Hyena*, Skunk*
Behav. 11			Horses, Horse, Cows*, Camel*, Yeehah animal*, Horse cart*, Water buffalo*
Behav. 12			Alligator, Crocodile, Lizard*
Behav. 13			Chipmunk, Squirrel, Rat, Mouse, Weasel
Behav. 14			Rooster, Cockerel, Chicken, Hen, Bird, Cock, Duck*
Behav. 15			Scissors, Knife*, Cutting*
Behav. 16			Cow, Cows, Ox, Moo*
Behav. 17			Woman, Lady, Girl, Person, Human, Face, Hat, Cowboy hat, Sombrero, Queen, Witch, Man*, Detective*
Behav. 18			Man, Boy, Face, Person, Human, Grandpa, Old lady, Cowboy Hat*, People*, Witch*, Mouth*
Behav. 19			Dog, Breed of dog, Puppy, Otter*
Behav. 20			Panda, Giant panda, Bear, Koala*

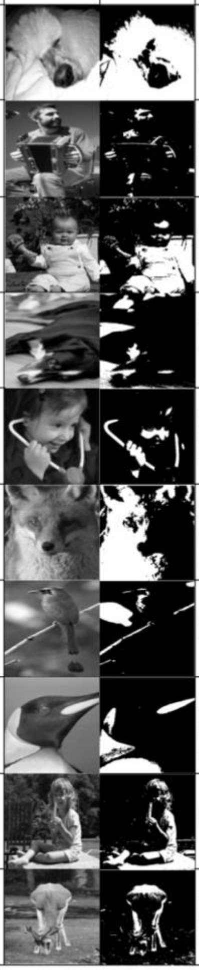

	Greyscale	Two-tone	Accepted Answers
Trained 1			Dog, Breed of dog
Trained 2			Man, Human, Person, Accordion
Trained 3			Baby, Toddler, Maraca
Trained 4			Dog, Breed of dog
Trained 5			Girl, Boy, Doctor, Stethoscope
Trained 6			Fox, Type of fox, Dog, Wolf, Type of Wolf
Trained 7			Bird, Type of bird
Trained 8			Penguin, King penguin, Bird, Type of bird
Trained 9			Girl, Boy, Child, Ice lolly*
Trained 10			Deer, Antelope, Gazelle, Ox

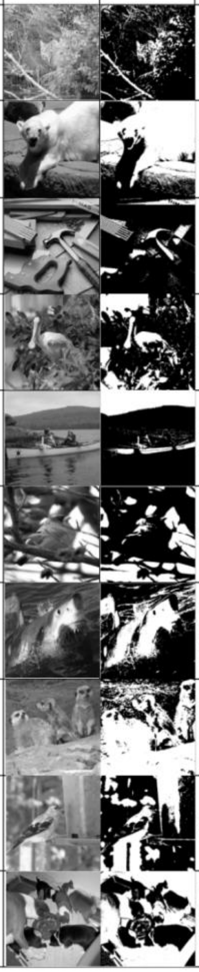

	Greyscale	Two-tone	Accepted Answers
Trained 11			Lynx, Cat, Big Cat, Snow Leopard
Trained 12			Polar bear, Ice bear, Bear
Trained 13			Hammer, Tools
Trained 14			Bird, Type of bird
Trained 15			Boat, Canoe, Paddle*
Trained 16			Bird, Type of bird
Trained 17			Shark, Great White, Whale, Killer Whale
Trained 18			Meerkat, Meerkats, Dog*, Dogs*, Type of Dog*
Trained 19			Bird, Type of bird
Trained 20			Dog, Dogs, Puppies, Breed of dog

Figure S2: Catch Trial Performance, *Related to Figures 1B and 2B.*

A) Naive naming accuracy of two-tones (coloured circles), and Catch images ('easy' two-tones created without smoothing; white circles). Small markers show participant means, large markers show age group means, error bars show bootstrapped 95% Confidence Intervals. **B)** Pointing accuracy for two-tones (coloured circles) and Catch trials (white circles) following greyscale exposure, as measured by percentage of touched locations falling within the predefined correct area for each target. Markers and error bars are as in Panel A. **C)** Catch trial performance of CNNs (greyscale bars) and participant age groups (coloured bars). Error bars show bootstrapped 95% Confidence Intervals for participant age groups.

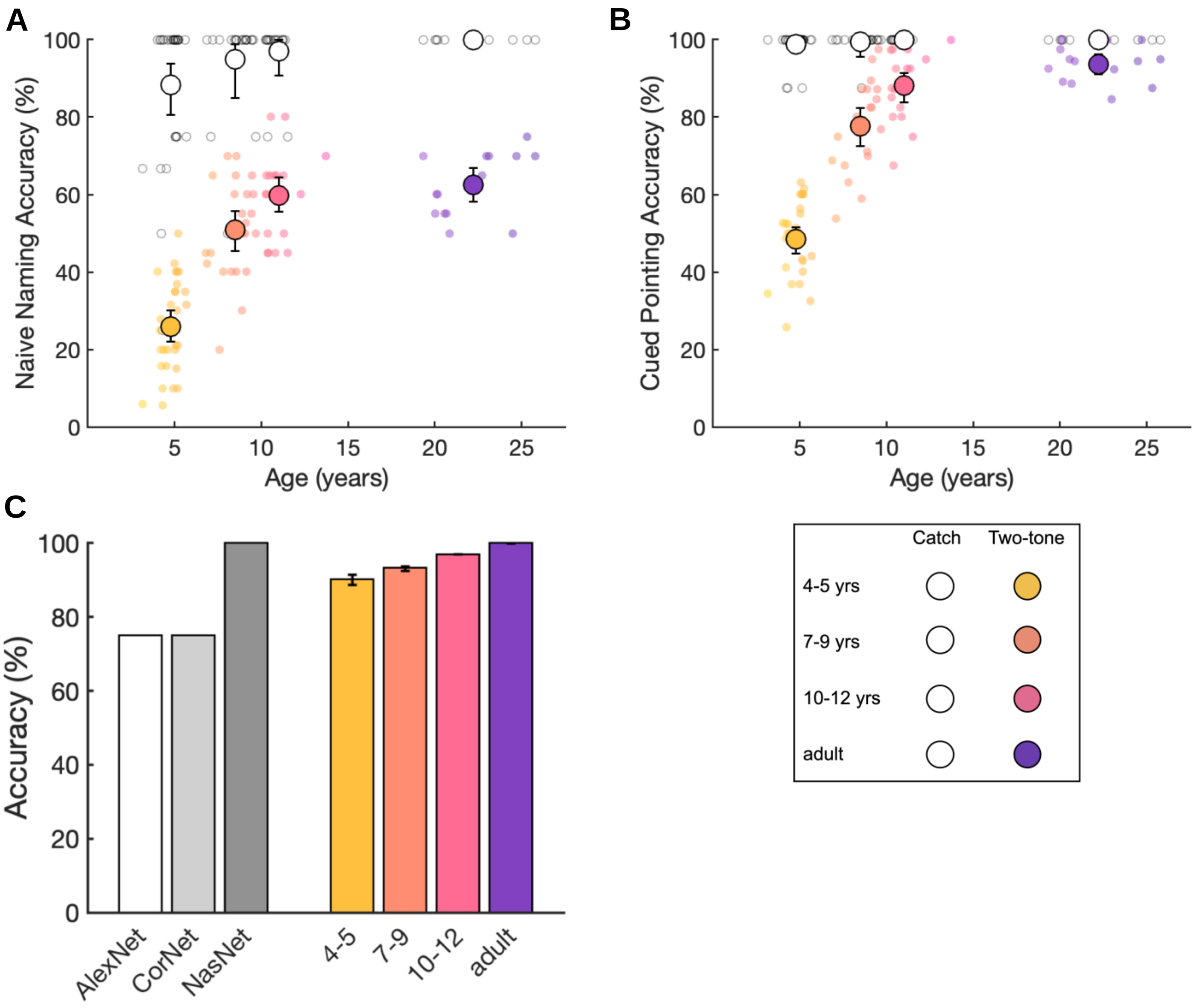

Figure S3: Feature Localisation Performance per Image.

Catch (top row) and two-tone images additional to those shown in Figure 4 (rows 2-5) with 4- to 5-year-olds' (yellow markers) and adults' (purple markers) cued two-tone pointing: target features 1 and 2 are shown in subsequent images. Target prompts were (1) 'the cup's handle', (2) 'the cup's saucer', (3) 'the frog's eye', (4) 'the frog's leg', (5) 'the middle of the flower', (6) 'the edge of one of the petals', (7) 'the rabbit's eye', (8) 'the rabbit's ear', (9) 'the left elephant's' tusk', (10) 'the right elephant's mouth', (11) 'the front of the train', (12) 'the column of the bridge', (13) 'the bear's left ear', (14) 'the bear's nose', (15) 'the koala's nose', (16) 'the koala's hand', (17) 'the left zebra's eye', (18) 'the right zebra's back leg', (19) 'the tiger's left ear', (20) 'the tiger's tail', (21) 'the fish's eye', (22) 'the fish's side fin', (23) 'the left horse's ears', (24) 'the right horse's nose', (25) 'the alligator's nose', (26) 'the alligator's eye', (27) 'the chipmunk's eye', (28) 'the chipmunk's tail', (29) 'the chicken's beak', (30) 'the chicken's eye', (31) 'the scissors' bolt', (32) 'the scissors' right handle', (33) 'the cow's nose', (34) 'the cow's rear', (35) 'the lady's nose', (36) 'the lady's hat', (37) 'the man's ear', (38) 'the man's nose', (39) 'the dalmatian's left eye' and (40) 'the dalmatian's nose'.

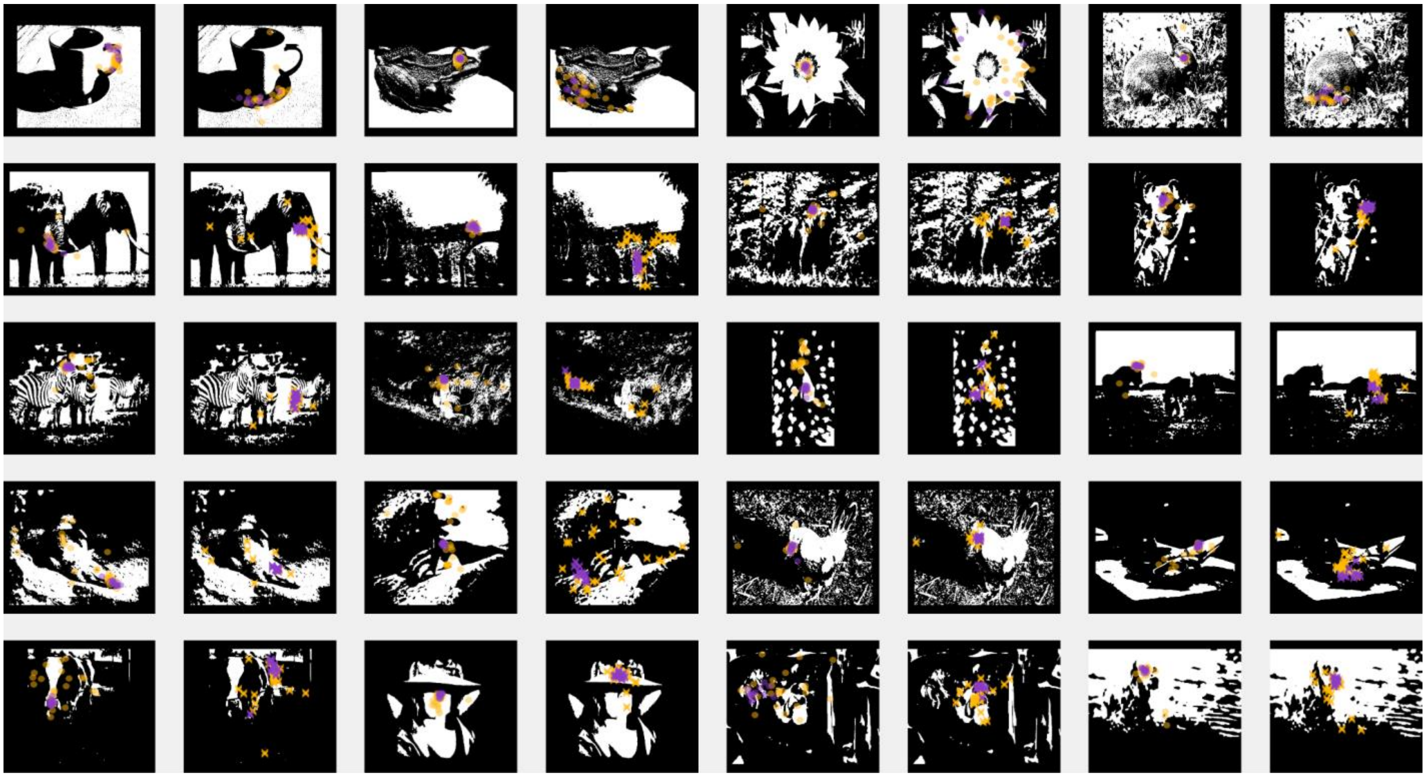

Figure S4: Image-wise correlations of human and CNN performance across conditions, *Related to Figures 1B, 2 and 4*.
Comparable results were found for Spearman Rank correlations.

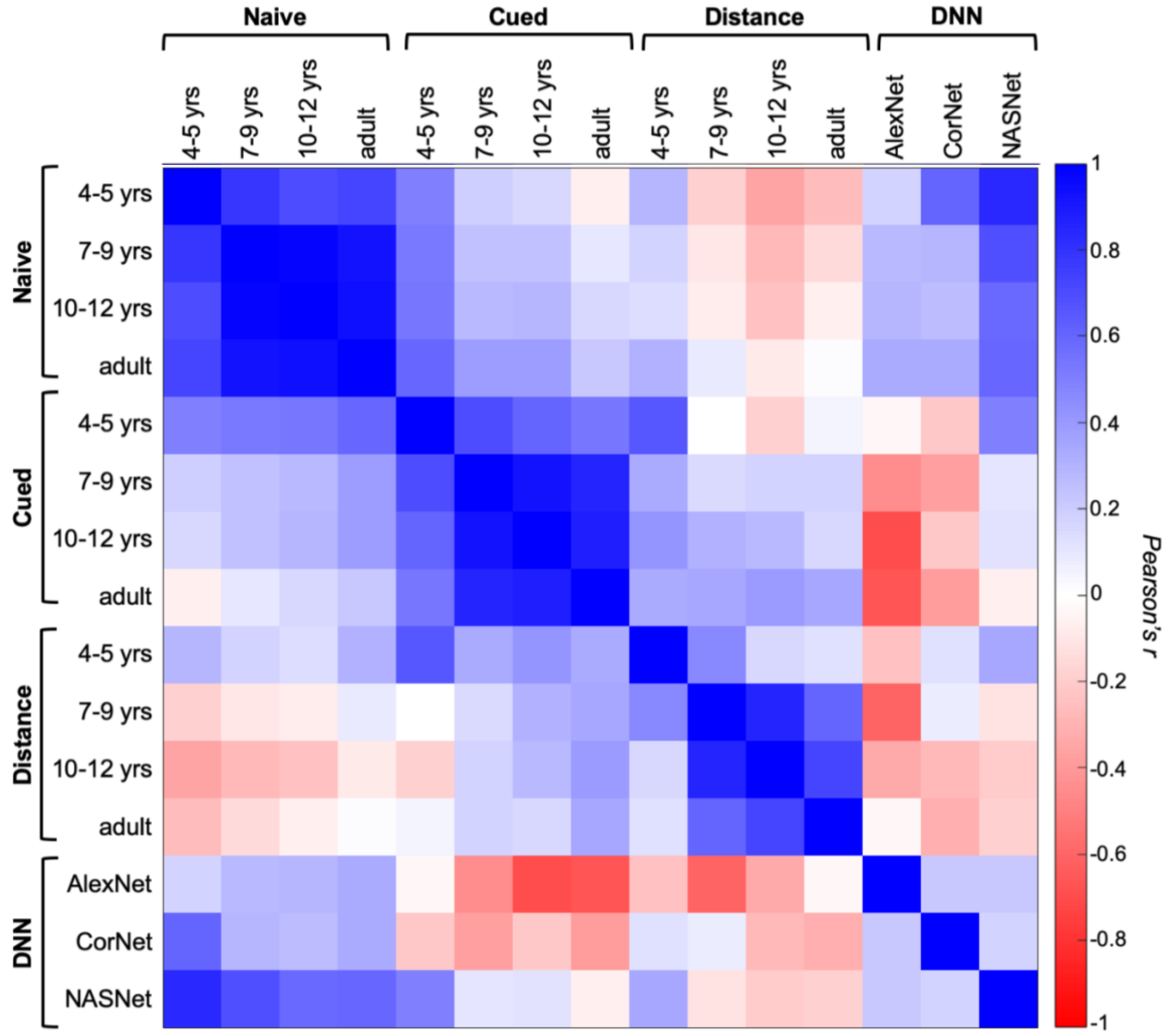

S5: Computational model for computing natural scene statistics, *Related to Figure 4*.

Low-level spatial information in the image such as edge and contrast metrics convey information about image content. This information has been found to guide parsing of objects in complex visual scenes[S1, S2]. In two-tone recognition, the disambiguation of obscured edges using object knowledge is thought to play an important role in the parsing process. We hypothesised that an image recognition strategy relying on spatial information about edges would be adversely affected by disruptions of this information following two-tone transformation. We tested this by quantifying several spatial image statistics for the greyscale and two-tone conditions of each image, as well as the change between these conditions. We predicted negative correlations between performance and the change in greyscale and two-tone image statistics, in particular for younger children who may rely more heavily on the image features rather than object knowledge.

Edge Density, the percentage of image pixels that represent edges, of the images in our dataset decreased following two-tone transformations. Larger reductions in Edge density imposed by converting from greyscale to two-tone correlated with poorer naive two-tone recognition in 4- to -5-year-olds' (see 'Δ Edge Density', Table S5). An overall higher two-tone Edge Density correlated with better Cued Accuracy for this age group (see 'Edge Density', Table S5).

We also computed biologically principled summary scene statistics using a gist extraction model that emulates how the magno- and parvocellular pathways process low-level features with neurally plausible image filtering[S3]. This model first convolves the image with a range of local edge filters that mimic parvo and magnocellular spatial filters[S4], the responses of which were rectified and divisively normalised following the LGN suppressive field approach[S5]. One filter per image location was selected using minimal reliable scale selection[S6], and the resulting spatially filtered images were then pooled into two summary statistics: Contrast Energy (CE) and Spatial Coherence (SC). To compute CE, the edge filter responses for the parvocellular pathway are averaged, resulting in a measure of mean image contrast, with high values reflecting more contrast. To compute Spatial Coherence (SC), the variability of edge filter responses is computed for the magnocellular pathway, resulting in a measure of contrast variability, with high values reflecting more contrast variations. In Groen et al.'s study[S3], these statistics were computed across a limited visual field area, to account for the fact that participants are fixating centrally on a briefly (100 ms) presented stimulus. However, in our study, images were free-viewed for extended periods, so we averaged statistics across the entire image (i.e., a 15-degree radius). Groen et al.[S3] showed that in adults, these model outputs predict perceived naturalness of the image and correlate with the EEG signal amplitude recorded across the occipital cortex whilst the image is categorised. We recently showed that this is also the case in children aged 6 years and upward, although less variance in the occipital EEG was explained by the model in the youngest children[S7].

Here, the conversion of greyscale images to two-tones was found to increase CE and reduce SC overall (see Figure S5). Greater alterations of these parameters corresponded with poorer Naive and Cued two-tone recognition in 4- to 5-year-olds, but no other age group (see Table S5: 'Δ CE'

and ‘Δ SC’). These data are tentatively in line with age-related reductions in the reliance on intact image statistics when object knowledge is available.

Table S5: Pearson’s correlations of image properties and performance by age group, *Related to Figure 4.*

Image properties are (1) Thresholding and (2) Smoothing levels used for the two-tone transformation of each image, (3) Edge Density, (4) Contrast Energy and (5) Spatial Coherence of resulting two-tone images, and Change between greyscale and two-tone images (Δ) for (6) Edge Density, (7) Contrast Energy and (8) Spatial Coherence. Correlations are uncorrected for multiple comparisons, and comparable results were found for Spearman Rank correlations.

		Naïve Naming Accuracy							Cued Pointing Accuracy			
		4-5 yrs	7-9 yrs	10-12 yrs	Adults	AlexNet	CorNet-S	NASNet	4-5 yrs	7-9 yrs	10-12 yrs	Adults
Threshold	Pearsons’ *r*	-0.529	-0.384	-0.293	-0.410	-0.036	-0.251	-0.469	-0.300	-0.306	-0.281	-0.055
	p	0.007**	0.064	0.164	0.048*	0.899	0.387	0.024*	0.154	0.145	0.183	0.797
Smoothing	Pearsons’ *r*	-0.695	-0.673	-0.611	-0.638	-0.498	-0.101	-0.625	-0.448	-0.442	-0.381	-0.162
	p	0.0002***	0.0003***	0.001**	0.0008***	0.059	0.731	0.001**	0.028*	0.031*	0.066	0.450
Edge Density	Pearsons’ *r*	0.389	0.382	0.360	0.371	0.414	0.052	0.467	0.467	0.316	0.275	0.185
	p	0.060	0.065	0.084	0.075	0.125	0.861	0.025*	0.021*	0.133	0.193	0.388
Contrast Energy	Pearsons’ *r*	0.525	0.317	0.249	0.343	-0.127	0.387	0.442	0.417	0.275	0.314	0.160
	p	0.008**	0.131	0.240	0.100	0.653	0.172	0.035*	0.043*	0.193	0.135	0.456
Spatial Coherence	Pearsons’ *r*	0.283	0.247	0.234	0.322	-0.031	0.200	0.350	0.306	0.108	0.113	0.005
	p	0.180	0.245	0.271	0.125	0.911	0.493	0.102	0.146	0.616	0.599	0.982
Δ Edge Density	Pearsons’ *r*	0.525	0.372	0.261	0.197	0.498	0.110	0.538	0.298	0.214	0.158	0.137
	p	0.008**	0.073	0.219	0.356	0.059	0.708	0.008**	0.157	0.315	0.460	0.524
Δ Contrast Energy	Pearsons’ *r*	0.6385	0.390	0.311	0.398	0.008	0.475	0.491	0.448	0.300	0.283	0.143
	p	0.0008***	0.060	0.140	0.054	0.977	0.086	0.017*	0.028*	0.155	0.181	0.506
Δ Spatial Coherence	Pearsons’ *r*	-0.454	-0.376	-0.329	-0.352	-0.367	-0.161	-0.581	-0.293	-0.015	0.090	0.101
	p	0.026*	0.070	0.117	0.091	0.178	0.583	0.004**	0.164	0.946	0.675	0.639

Figure S5: Two-tone and greyscale differences in image statistics, *Related to Figure 4.*
A) Contrast Energy and Spatial Coherence of greyscale (blue borders), two-tone (pink borders) and catch (yellow borders) images. **B)** Comparison of average Edge Density, Contrast Energy and Spatial Coherence for greyscale, catch and two-tone stimuli. Error bars show bootstrapped 95% Confidence Intervals.

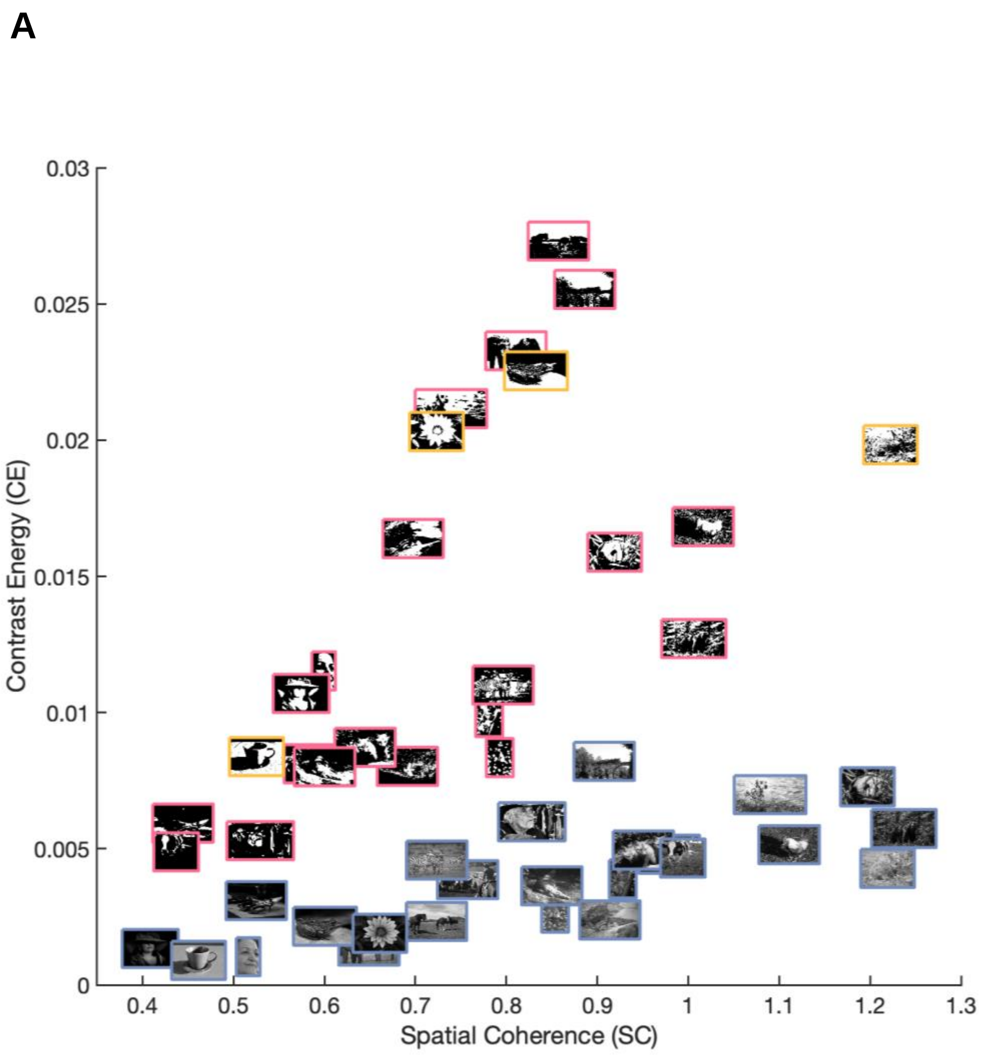

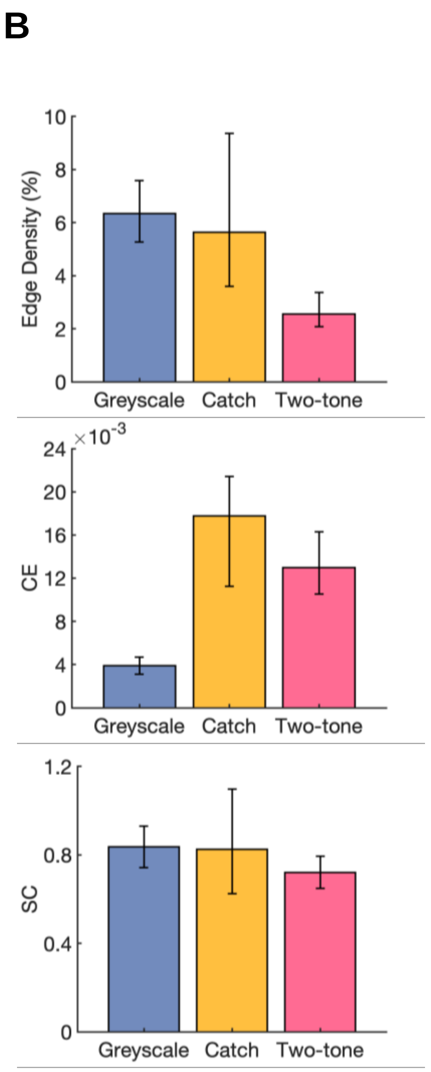

Supplemental References

S1. Groen, I.I.A., Jahfari, S., Seijdel, N., Ghebreab, S., Lamme, V.A.F., Scholte, H.S., 2018. Scene complexity modulates degree of feedback activity during object detection in natural scenes. PLOS Computational Biology 14, e1006690. doi.org/10.1371/journal.pcbi.1006690

S2. Seijdel, N., Jahfari, S., Groen, I.I.A., Scholte, H.S., 2020. Low-level image statistics in natural scenes influence perceptual decision-making. Sci Rep 10, 10573. doi.org/10.1038/s41598-020-67661-8

S3. Groen, I.I.A., Ghebreab, S., Prins, H., Lamme, V.A.F., Scholte, H.S., 2013. From Image Statistics to Scene Gist: Evoked Neural Activity Reveals Transition from Low-Level Natural Image Structure to Scene Category. Journal of Neuroscience 33, 18814–18824. doi.org/10.1523/JNEUROSCI.3128-13.2013

S4. Scholte, H.S., Ghebreab, S., Waldorp, L., Smeulders, A.W.M., Lamme, V.A.F., 2009. Brain responses strongly correlate with Weibull image statistics when processing natural images. Journal of Vision 9, 29–29. doi.org/10.1167/9.4.29

S5. Bonin, V., Mante, V., Carandini, M., 2005. The Suppressive Field of Neurons in Lateral Geniculate Nucleus. Journal of Neuroscience 25, 10844–10856. doi.org/10.1523/JNEUROSCI.3562-05.2005

S6. Ghebreab, S., Smeulders, A.W.M., Schoite, H.S., Lamme, V.A.F., 2009. A Biologically Plausible Model for Rapid Natural Image Identification 9. Presented in Advances in Neural Information Processing Systems 22 (NIPS 2009). ISBN: 9781615679119

S7. Chow-Wing-Bom, H.T., Scholte, S., De Klerk, C., Mareschal, D., Groen, I.A., Dekker, T., 2019. Development of Rapid Extraction of Scene Gist. Presented at the 42nd European Conference on Visual Perception (ECVP) 2019 Leuven. Perception, 48, 2, 1–236. doi.org/10.1177/0301006619863862
